# Exploring the interconnected between type 2 diabetes mellitus and nonalcoholic fatty liver disease: Genetic correlation and Mendelian randomization analysis

**DOI:** 10.1097/MD.0000000000038008

**Published:** 2024-05-10

**Authors:** Wenjuan Ni, Yao Lu, Wei Wang

**Affiliations:** a Department of Endocrinology, First Affiliated Hospital of Baotou Medical Collage, Baotou, Inner Mongolia, China; b Baotou Medical Collage, Baotou, Inner Mongolia, China.

**Keywords:** GWAS, Mendelian randomization, nonalcoholic fatty liver disease, shared genetics, type 2 diabetes mellitus

## Abstract

Epidemiological and clinical studies have indicated a higher risk of nonalcoholic fatty liver disease (NAFLD) and type 2 diabetes mellitus (T2DM), implying a potentially shared genetic etiology, which is still less explored. Genetic links between T2DM and NAFLD were assessed using linkage disequilibrium score regression and pleiotropic analysis under composite null hypothesis. European GWAS data have identified shared genes, whereas SNP-level pleiotropic analysis under composite null hypothesis has explored pleiotropic loci. generalized gene-set analysis of GWAS data determines pleiotropic pathways and tissue enrichment using eQTL mapping to identify associated genes. Mendelian randomization analysis was used to investigate the causal relationship between NAFLD and T2DM. Linkage disequilibrium score regression analysis revealed a strong genetic correlation between T2DM and NAFLD, and identified 24 pleiotropic loci. These single-nucleotide polymorphisms are primarily involved in biosynthetic regulation, RNA biosynthesis, and pancreatic development. generalized gene-set analysis of GWAS data analysis revealed significant enrichment in multiple brain tissues. Gene mapping using these 3 methods led to the identification of numerous pleiotropic genes, with differences observed in liver and kidney tissues. These genes were mainly enriched in pancreas, brain, and liver tissues. The Mendelian randomization method indicated a significantly positive unidirectional causal relationship between T2DM and NAFLD. Our study identified a shared genetic structure between NAFLD and T2DM, providing new insights into the genetic pathogenesis and mechanisms of NAFLD and T2DM comorbidities.

## 1. Introduction

Type 2 diabetes mellitus (T2DM) is characterized by chronic hyperglycemia that evolves from insulin resistance (IR) and beta cell dysfunction. In its natural course, insulin secretion increases temporarily, accompanied by secondary IR and progressive loss of beta cell mass.^[1]^ Metabolic disturbances accompanying obesity and dysfunction of the musculo–hepatopancreatic axis lead to the development of T2DM.^[2]^ Nonalcoholic fatty liver disease (NAFLD) occurs when the fat exceeds 10% to 5% of the weight of the liver. One of the most common liver diseases is an emerging public health problem issue worldwide.^[3]^ Obesity and physical inactivity are interlinked risk factors for the development of diabetes NAFLD.^[4]^

NAFLD is strongly associated with the risk of developing diabetes. The risk of developing diabetes is approximately 5-fold higher in patients with NAFLD.^[5,6]^ Estimates for the coexistence of NAFLD and T2DM vary, ranging from 30% to 80% of patients (average, 55.5%). An Italian study found a 70% prevalence of NAFLD in T2DM patients, while recent studies reported a range of 34% to 94%.^[7]^ This association could be explained by IR, dyslipidemia, hepatic TG accumulation in NAFLD, and defective B-cell in T2DM.^[8]^ IR leads to a dysfunction in adipokine production. This results in a proinflammatory state and increased oxidative stress due to the formation of reacting oxygen species. This causes the oxidation of free fatty acids and enhanced de novo lipogenesis, leading to the accumulation of TG.^[9]^ NAFLD and IR are bidirectionally correlated and, consequently, the development of prediabetes and diabetes is the most direct consequence at the extrahepatic level.^[10]^ In turn, T2DM is a well-known risk factor for multiorgan damage and T2DM patients have a higher risk of developing advanced liver disease.^[11]^ However, the association between NAFLD and T2DM remains to be elucidated. Some data suggest that NAFLD may predict T2DM and vice versa. Patients with diabetes and NAFLD have poorer glycemic control and more complications.^[12]^ Similarly, Excessive fatty liver infiltration in patients with T2DM can worsen NAFLD.^[13]^ Numerous epidemiological studies have confirmed the link between liver fat infiltration, IR, and T2DM, emphasizing the need for the diagnosis of both conditions in patients with NAFLD and T2DM.^[14]^ Moreover, identifying patients with both NAFLD and T2DM is crucial because of the mutual impact of these pathologies on the progression and appearance of life-threatening complications.^[15]^ Among the existing therapeutic drugs, Pioglitazone appears to be beneficial for patients with nonalcoholic steatohepatitis (NASH), making it the only drug approved by all major liver societies for NASH accompanied by significant liver fibrosis. Conversely, sodium-glucose transport protein-2 inhibitors inhibitors and glucagon-like peptide 1 receptor analogs seem to offer advantages for patients with NAFLD, displaying impressive results. In the only head-to-head study to date, SGLT2 inhibitors have proven to be more efficient than GLP1 receptor agonists.^[16]^ However, there is currently no clear target for the treatment of comorbidities, and no drugs have been approved or applied in the clinic for both diseases.

Genome-wide association studies (GWAS) have identified multiple genetic variants (i.e., single-nucleotide variations; formerly single-nucleotide polymorphisms [SNPs]) associated with T2DM and NAFLD. Genetic correlations between these diseases have been suggested using linkage disequilibrium score regression (LDSC). However, the specific loci involved remains unclear.^[17]^ It is crucial to identify these genomic variants and explore the shared genetic etiology of T2DM and NAFLD.

In this genome-wide pleiotropic study, we analyzed T2DM and NAFLD using the GWAS summary data. We sequentially investigated pleiotropic associations at genome-wide, gene, and biological pathway levels. We detected pleiotropic variants and loci at the SNV level, identified candidate genes, and characterized their phenotype and tissue specificity. Additionally, we performed Mendelian randomization analysis to assess causal associations and pleiotropy types.

## 2. Materials and methods

### 2.1. Study design

To evaluate the complex genetic relationship between T2DM and NAFLD, the genetic correlation between T2DM and NAFLD was evaluated using the LDSC method, the corresponding pleiotropic loci and genes were identified using the pleiotropic method, and the causal relationship between them was evaluated using the two-sample bidirectional Mendelian randomization (MR) method.

**Figure 1.**
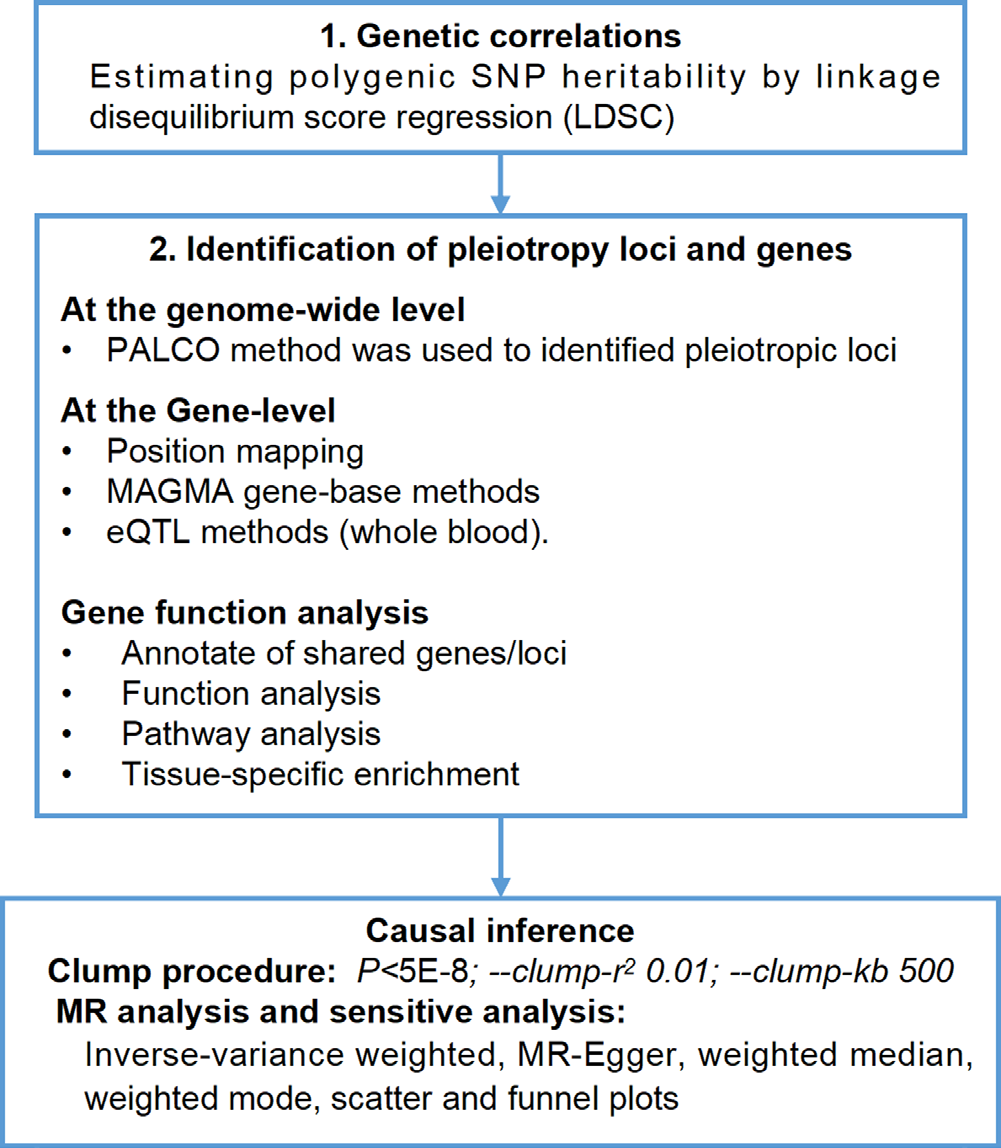
Study overview.

The overall study design is presented in Figure 1.

### 2.2. Data sources and instruments

#### 2.2.1. T2DM data

T2DM was obtained from the Meta-analysis Alliance of Diabetes Meta-Analysis of Trans-Ethnic Association Studies Consortium. In this study, 180,834 cases and 1159,055 controls (51.1% of European origin) were analyzed using GWAS. The association summary statistics of GWAS were integrated through the meta-analysis of fixed effects and random effects using METAL software and METASOFT, respectively. Both meta-analyses were based on inverse variance weighting of the allele logarithm OR to estimate effect size. We extracted only the European sample data for the analysis.

#### 2.2.2. NAFLD data

NAFLD data were collected from Finngen R9gwas (https://r9.finngen.fi/), including 2275 cases and 375002 controls. The model included the following covariables: sex, age, 10 genetic principal components, and genotyping batches. FinnGen collected and analyzed the genome and health data of 500,000 participants in the Finnish biological sample bank. On the 1 hand, it provides novel medical and therapeutic insights and, at the same time, builds world-class resources that can be used for future research.

### 2.3. Genetic correlation analysis

The LDSC method^[18]^ was used to evaluate the shared polygene structure among traits, in which the LD score in LDSC was calculated from the thousand-person genome as a reference group and the European lineage samples in the Hapmap3 project.^[19]^ For SNP, we implemented strict quality control: (i) excluding non-biallelic SNPs and those with chain fuzzy alleles; (ii) excluding SNP; without rs tags; (iii) deletion of repeated SNPs or SNPs that are not included in the 1000 genome project or whose alleles do not match; (iv) SNP located in the major histocompatibility complex (chr6:28.5–33.5Mb) was excluded from the LDSC analysis because of its complex LD structure; (v) SNP with minor allele frequency (MAF) > 0.01.

### 2.4. Pleiotropic analysis under composite null hypothesis (PLACO)

*SNP-level* PLACO is a new method that can only use the statistical data of genotype-phenotype associations at the aggregate level to study pleiotropic loci among complex traits.^[20]^ We calculated the square of the Z score for each variant and removed SNP with extremely high Z2 values (>80). In addition, considering the potential correlation between T2DM and NAFLD, we estimated the correlation matrix for Z. Subsequently, the horizontal α cross-joint test method was used to test the pleiotropic hypothesis. The final *P*-value of the horizontal α cross-joint test was the maximum *P*-value of tests H0 and H1.

Based on the PLACO results, we mapped the identified loci to nearby genes, and discussed the common biological mechanisms of these pleiotropic loci. To identify candidate pleiotropic pathways and tissue enrichment of pleiotropic genes, we conducted a Generalized Gene-Set Analysis of GWAS Data (MAGMA)^[21]^ based on PLACO output and single trait GWAS. Functional Mapping and Annotation of Genome-Wide Association Studies were used to determine the biological functions of pleiotropic loci.^[22]^ Based on the molecular feature database, a series of enrichment analysis methods was used to determine the function of the mapped gene.^[23]^ Notably, this part of the MAGMA gene set and tissue-specific analysis used the complete distribution of SNP p-values. eQTL analysis included SNP-gene association data for whole blood tissue.

### 2.5. Mendel randomization study

We used the clumping program^[24]^ in PLINK software to screen out all the significant gene loci independently related to diseases as instrumental variables (T2DM: *P* < 5 × 10^−8^), The *r*^2^ threshold of instrumental variables was set to 0.001, and the window was set to 10,000 kb. To ensure the strength of the tool variables, we calculated *r*^2^ and *F* statistics for each tool variable.^[25]^ The F-statistic calculation formula is as follows: $F=\left(\frac{n-z-k}{k}\right)\left(\frac{r^{2}}{1-r^{2}}\right)$, where *r*^2^ represents the variance ratio explained by the tool variables, *n* represents the sample size, and *k* represents the number of SNP. The main method of Mendel randomization is the inverse variance weighting (IVW) method, which requires the instrumental variable (IV) to meet 3 assumptions: (i) IV should be related to exposure; (ii) IV should not be associated with confounding factors associated with exposure and results; and (iii) the influence of IV on the results is completely achieved through exposure. Several sensitivity analyses were performed. First, the Q-test using IVW and MR-Egger can detect the potential violation hypothesis through the heterogeneity of the association between IV.^[26]^ Second, we used MR-Egger to estimate horizontal pleiotropy according to its intercept to ensure that genetic variation is independently related to exposure and results.^[27]^ The global MR-PRESSO test was also used to evaluate potential horizontal pleiotropy. We increased the stability and robustness of the results by using an additional analysis (weighted median and weighted mode) of MR methods with different modeling assumptions and advantages.

Statistical analysis was carried out using R3.5.3, and MR analysis was performed using the Mendelian Randomization software package.^[28]^

## 3. Results

### 3.1. Genetic correlation

Genetic correlation analysis of LDSC revealed a significant genetic correlation between T2DM and NAFLD (Table S1, Supplemental Digital Content, http://links.lww.com/MD/M377), whether in the model with the intercept term (*R* = 0.727, *P* = 2.01E−06) or in the model without the intercept term (*R* = 0.535, *P* = 1.17E−22).

### 3.2. Identification of pleiotropic loci

**Figure 2.**
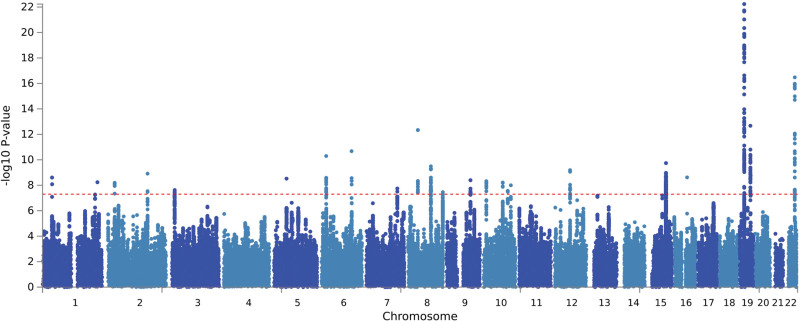
Manhattan map of pleiotropic loci between T2DM and NAFLD. NAFLD = nonalcoholic fatty liver, T2DM = type 2 diabetes mellitus.

PLACO pleiotropic analysis was performed for both diseases. The Manhattan map is shown in Figure 2 and the pleiotropic loci identified are listed in Table 1. Twenty-four pleiotropic loci were identified. No genomic inflation was found in the QQ map (Fig. S1, Supplemental Digital Content, http://links.lww.com/MD/M383), the basic information of each genome risk locus is shown in Figure S2, Supplemental Digital Content, http://links.lww.com/MD/M384; Effects of pleiotropic SNP on gene function include intronic, intergenic, ncRNA-intronic, UTR3, upstream, and TUR5. The results are shown in Figure S3, Supplemental Digital Content, http://links.lww.com/MD/M385.

**Table 1:** Information of 24 pleiotropic loci identified.

Genomic locus	uniqID	Lead SNPs	chr	Start	End	*P*	nSNPs	nGWASSNPs	Mapped genes
1	1:39628880:A:G	rs143026914	1	39521386	40107757	2.47E−09	431	111	MACF1
2	1:229672955:A:G	rs348330	1	229531880	229982892	5.79E−09	377	251	ABCB10
3	2:27734972:A:G	rs6547692	2	26754960	28597624	6.34E−09	668	376	GCKR
4	2:165539661:C:T	rs6717858	2	165319311	165826943	1.22E−09	377	239	GRB14, COBLL1
5	3:12216260:A:G	rs6781687	3	11973918	12756379	2.39E−08	289	160	SYN2
6	5:53285762:A:T	rs114118094	5	53215804	53593719	3.02E−09	103	61	ARL15
7	6:20536992:A:G	rs2143407	6	20383121	21132036	5.06E−11	877	396	CDKAL1
8	6:126792095:A:G	rs11759026	6	126623947	127385376	2.10E−11	524	317	C6orf173, MIRN588
9	7:130447130:A:G	rs59326756	7	130350281	130468190	1.80E−08	104	72	KLF14, LOC347674
10	8:41481037:G:T	rs890222	8	41192234	41630744	4.55E−13	549	330	AGPAT6, NKX6-3
11	8:95968413:G:T	rs726816	8	95286065	96200766	3.24E−10	746	494	TP53INP1, C8orf38
12	8:145903077:A:C	rs997258	8	145639320	146301338	3.47E−08	507	278	KIAA1688, ZNF251
13	9:100952197:C:T	rs112052630	9	100922202	101066913	4.01E−09	124	71	
14	10:12269343:C:T	rs11257608	10	12014080	12410160	4.76E−09	302	186	CDC123
15	10:80964796:A:C	rs1250576	10	80906729	81069413	6.08E−09	174	99	ZMIZ1
16	10:101924418:C:T	rs10883451	10	101522170	102078594	2.72E−08	362	178	ERLIN1
17	10:114891443:A:G	rs11599737	10	114723383	114920089	9.99E−09	74	41	TCF7L2
18	12:66166276:C:T	rs12303535	12	66138808	66408293	6.49E−10	189	110	LOC204010, HMGA2
19	15:77892857:A:G	rs58102377	15	77334787	77992898	1.82E−10	601	321	HMG20A, LINGO1
20	16:53817318:A:G	rs113191842	16	53755146	53848561	2.41E−09	150	9	FTO
21	19:19419071:A:G	rs739846	19	19260760	19989679	5.85E−23	417	309	SF4
22	19:45411941:C:T	rs429358	19	45376893	45429708	2.12E−13	51	36	APOE/LOC100129500
23	19:46157004:C:T	rs10408179	19	46089212	46378515	1.74E−09	406	198	MIRN330, GIPR
24	22:44382684:C:T	rs2294927	22	44323597	44412192	3.33E−17	281	149	SAMM50

The results of pleiotropy were analyzed by MAGMA gene set enrichment, and the results showed that the top 5 significant gene sets enriched were: go pancreas development, go beta-catenin TCF complex, go positive regulation of biosynthetic process, go positive regulation of RNA biosynthetic process, and go positive regulation of gene expression (Table 2). Tissue-specific MAGMA analysis showed that these 2 diseases were significantly enriched in multiple brain tissues (Fig. S4, Supplemental Digital Content, http://links.lww.com/MD/M386 and Table S2, Supplemental Digital Content, http://links.lww.com/MD/M378).

**Table 2:** Gene set analysis results (top 10).

Gene set	N genes	Beta	SE	*P*
GO bp: go pancreas development	76	0.494	0.105	1.21E−06
GO cc: go beta catenin tcf complex	11	1.249	0.284	5.42E−06
GO bp: go positive regulation of biosynthetic process	1847	0.089	0.021	1.70E−05
GO bp: go positive regulation of rna biosynthetic process	1509	0.097	0.024	2.13E−05
GO bp: go positive regulation of gene expression	1828	0.087	0.022	3.24E−05
GO mf: go insulin binding	5	1.909	0.502	7.11E−05
GO bp: go negative regulation of gene silencing	31	0.576	0.154	8.94E−05
Curated gene sets: KEGG adherens junction	70	0.439	0.118	9.96E−05
GO bp: go regulation of carbohydrate catabolic process	78	0.370	0.100	1.06E−04
Curated gene sets: reactome regulation of gene expression in late stage branching morphogenesis pancreatic bud precursor cells	15	0.869	0.238	1.33E−04

### 3.3. Identification of pleiotropic genes

Using the position information of the lead SNP, we matched 33 genes related to these pleiotropic risk loci (Table 1, last column). Some pleiotropic genes were obviously differentially expressed in liver and kidney tissues, such as GRB14, At the same time, GCKR was only significantly overexpressed in liver tissue (Fig. S5, Supplemental Digital Content, http://links.lww.com/MD/M387). Through tissue-specific enrichment analysis, it was revealed that the genes mapped to these positions were located in various tissues including abdominal muscles, brain, pancreas, and others. (Fig. S6, Supplemental Digital Content, http://links.lww.com/MD/M388).

Pathway enrichment analysis showed enrichment in the regulation of developmental growth, response to hexose, regulation of the Wnt signaling pathway, regulation of small molecule metabolism, process positive regulation of cellular, and catabolic process glutamatergic synapses (Fig. S7, Supplemental Digital Content, http://links.lww.com/MD/M389). Cell type enrichment analysis showed enrichment in lake adult kidney C20 collecting duct inter-calated cells type a cortex, lake adult kidney C14 distal convoluted tubule, and Zhong pfc major types of astrocytes (Fig. S8, Supplemental Digital Content, http://links.lww.com/MD/M390).

**Figure 3.**
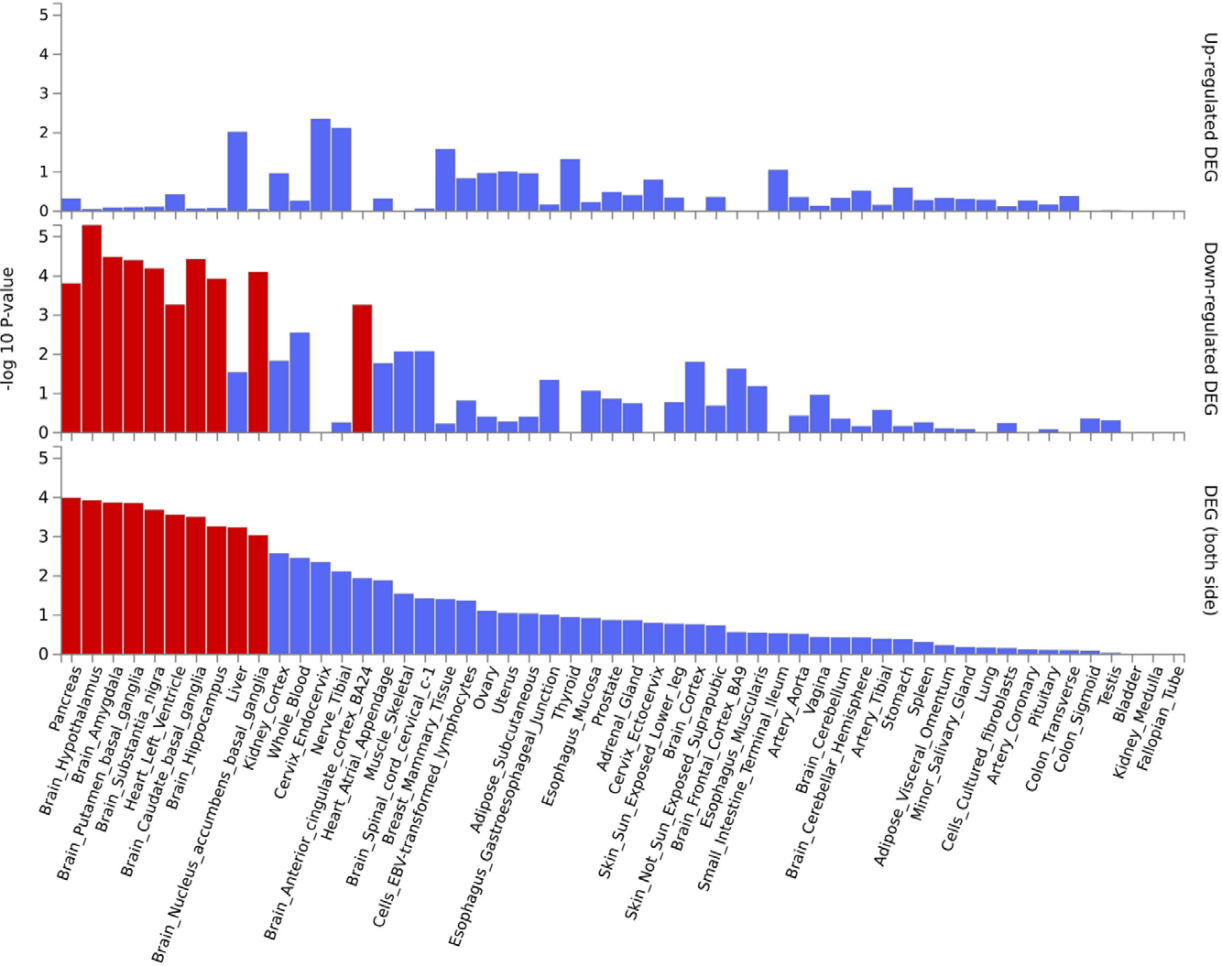
Expression of pleiotropic genes in different tissues.

Based on the Magma test, we identified 27 pleiotropic genes (*P* < .05, 18645 = 2.682E−6) (Table S3, Supplemental Digital Content, http://links.lww.com/MD/M379 and Fig. S9, Supplemental Digital Content, http://links.lww.com/MD/M391). Some pleiotropic genes also showed obvious differential expression in the liver and kidney tissues, such as transmembrane 6 superfamily 2 (TM6SF2), which was highly expressed only in the liver, moderately expressed in the kidney and spleen, and weakly expressed in other tissues (Fig. S10, Supplemental Digital Content, http://links.lww.com/MD/M392). Tissue-specific enrichment analysis showed that these mapped genes were enriched in the pancreas, brain, liver, and other tissues (Fig. 3). Pathway enrichment analysis showed enrichment in triglyceride (TG) metabolic processes, catalytic activity, acting on DNA fat cell differentiation, cellular response to organic cyclic compounds, extracellular matrix, chromatin remodeling, regulation of secretion, and membrane organization (Fig. S11, Supplemental Digital Content, http://links.lww.com/MD/M393). The cell type enrichment analysis showed enrichment in Travaglini lung macrophage cells, human fetal retina rpe, Rubenstein skeletal muscle satellite cells, Zhong pfc major-type astrocytes, fan embryonic ctx big groups microglia, Zhong pfc major type microglia, mannomidbrain neurotypes hrgl2b, aizaraniliver C30 hepatocytes 4, and descartes fetal liver hepatoblasts (Fig. S12, Supplemental Digital Content, http://links.lww.com/MD/M394).

Using eQTL information (whole blood tissue), we matched eQTL genes (Tables S4, Supplemental Digital Content, http://links.lww.com/MD/M380 and S5, Supplemental Digital Content, http://links.lww.com/MD/M381) related to these pleiotropic risk loci. Some pleiotropic genes such as ABCC2, KHK, DHTKD1and DMWD also showed obvious differential expression in liver and kidney tissues (Fig. S13, Supplemental Digital Content, http://links.lww.com/MD/M395). Tissue-specific enrichment analysis showed that these mapped genes were enriched in tissues, such as the pancreas, brain, liver, and whole blood (Fig. S14, Supplemental Digital Content, http://links.lww.com/MD/M396). Pathway enrichment analysis showed enrichment in nuclear organization, hsa03015:mRNA surveillance pathway, membrane organization, cellular respiration, phosphotransferase activity, alcohol group as acceptor, response to salt WP411:mRNA processing, focal adhesion, regulation of small GTPase-mediated signal transduction, chromosome organization, primary active transmembrane transporter activity, cellular nitrogen compound catabolic process, and hsa05168: herpes simplex virus 1 infection (Fig. S15, Supplemental Digital Content, http://links.lww.com/MD/M397). Cell type enrichment analysis showed enrichment in Travaglini lung B cells, Travaglini lung proximal basal cells, and fan ovarycl8 mature cumulus granulosa cell 2 (Fig. S16, Supplemental Digital Content, http://links.lww.com/MD/M398).

### 3.4. Causal associations between T2DM and NAFLD

**Figure 4.**
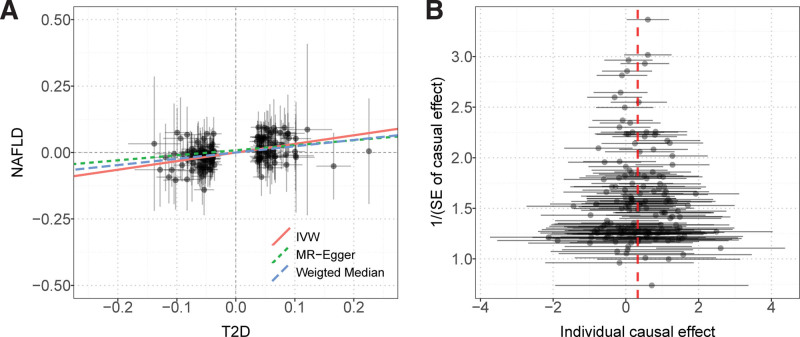
Scatter diagram and funnel diagram of MR analysis, A is the scatter plot of the causal effect of T2DM on NAFLD; B is the funnel diagram of the causal effect of T2DM on NAFLD. MR = Mendelian randomization, T2DM = type 2 diabetes mellitus, NAFLD = nonalcoholic fatty liver.

Finally, the causal relationship between the 2 diseases was inferred using a two-sample MR method, and the results supported a significant causal relationship between T2DM and NAFLD. The causal effect analysis of T2DM on NAFLD showed that the 3 MR methods (IVW, weighted median, and MR-PRESSO) presented consistent results and that T2DM increased the risk of NAFLD. See Table S6, Supplemental Digital Content, http://links.lww.com/MD/M382 for the tool variables. The sensitivity analysis is shown in Table 3, and the heterogeneity test *P* value was .037, which is heterogeneous; therefore, the IVW method with random effects was selected for inference. The intercept term *P* > .05 for MR-Egger and the global test (*P* > .05) of MR-PRESSO ruled out possible horizontal pleiotropy in causal inference. Scatter and funnel plots are shown in Figure 4, and no outlier interference was observed.

**Table 3:** MR analysis results.

Exposure	Outcome	Methods	OR (95%CI)	*P*	Heterogeneity test	
					Estimate	*P*
T2D	NAFLD	IVW (fixed)	1.383 (1.259, 1.52)	1.52E−11	190.119	.037
		IVW (random)	1.383 (1.247, 1.534)	8.75E−10		
		MR-Egger (slope)	1.208 (0.884, 1.651)	.233		
		MR-Egger (intercept)	0.008 (-0.01, 0.027)	.365		
		Weighted mode	1.077 (0.78, 1.487)	.654		
		Weighted median	1.266 (1.093, 1.467)	.002		
		MR-PRESSO	1.383 (1.247, 1.534)	6.80E−09		
NAFLD	T2D	IVW (fixed)	1.028 (0.972, 1.086)	.334	1.385	.239
		IVW (random)	1.028 (0.963, 1.097)	.412		

For NAFLD, only 2 instrumental variables were screened in this study; therefore, further sensitivity analyses could not be performed. Simultaneously, the possibility of a reverse causal effect of NAFLD on T2DM is ruled out.

## 4. Discussion

The global prevalence of diabetes has continued to increase. According to the latest report from the International Diabetes Federation, 10.5% of the world’s adult population have diabetes. If these trends continue, 1 in 8 adults will be living with diabetes by 2045.^[29]^ Similarly, NAFLD affects one-quarter of the global population.^[30]^ The study of T2DM has been driven by advances in human genetics, epigenetics, biomarkers, mechanistic studies, and large clinical trials, providing new insights into disease susceptibility, pathophysiology, progression, development of complications, and their complex relationship with NAFLD. The pathogenesis of T2DM and NAFLD is intricate and not fully understood. However, many factors are also involved in their development. These include genetic predispositions,^[31,32]^ environmental and lifestyle influences,^[33]^ intestinal microbiome alterations,^[34,35]^ IR,^[36]^ and disturbances in lipid and glucose metabolisms.^[37]^ The development of NAFLD is strongly associated with hepatic IR.^[38]^ This predisposes individuals to compensatory hyperinsulinemia, pancreatic cell dysfunction, or T2DM.^[39]^ Mathematical modeling reveals that NASH patients with T2DM will contribute to 1.27 million decompensated cirrhosis person-years, 479,000 hepatocellular carcinoma person-years, 29% of liver transplants, 812,000 liver-related deaths, and 1.37 million cardiovascular deaths over the next 2 decades.^[40]^ These alarming projections demand a comprehensive public health response to tackle this global health crisis, involving not only hepatologists but also other clinicians, particularly primary care physicians, diabetes care providers, and endocrinologists.^[41]^

The LDSC genetic correlation analysis revealed a significant genetic correlation between T2DM and NAFLD. PLACO pleiotropic analysis identified 24 pleiotropic loci for the 2 diseases, and tissue-specific MAGMA analysis revealed a significant enrichment in multiple brain tissues. The position information of the lead SNPs, MAGMA gene test, and eQTL information were used to identify and match the pleiotropic genes. All 3 methods demonstrated that some pleiotropic genes, such as GRB14, TM6SF2, and ABCC2, were significantly differentially expressed in liver and kidney tissues. MR showed that T2DM increased the risk of NAFLD, whereas NAFLD had no reverse causal effect on T2DM. By matching the genes related to these pleiotropic risk loci, we found that some pleiotropic genes such as GRB14, TM6SF2, and ABCC2 were differentially expressed in the liver.

Molecular adapter growth factor receptor-binding protein 14 (Grb14), a growth factor receptor-binding protein that is highly expressed in the liver, inhibits the catalytic activity of insulin receptors. Inhibition of Grb14 enhances insulin signaling, leading to the transient induction of S-phase entry by quiescent hepatocytes, suggesting that Grb14 is a potent repressor of cell division. The proliferation of Grb14-deficient hepatocytes is cell-autonomous, as observed in primary cell cultures.^[42]^ Data from human genetic studies have revealed that Grb14 acts as a negative regulator of IR activity, and germline Grb14-knockout mice exhibit enhanced insulin signaling in the liver and skeletal muscle. Grb14 knockdown in the liver, white adipose tissue, and heart using AAV-shRNA (Grb14-shRNA) improved glucose homeostasis in diet-induced obese mice.^[43]^ The human TM6SF2 variant rs58542926 is associated with NAFLD and hepatocellular carcinoma.^[44]^ It separates HCL from IR in recent-onset T2DM, an effect that is mitigated by the disease duration. GWAS found consistent associations between gene variants, such as TM6SF2, and NAFLD, offering new insights and potential drug targets.^[45]^ This suggests that metabolic changes related to diabetes override the effects of the TM6SF2 variant in the early stages of diabetes and NAFLD.^[46]^ Hepatic IR is a key mechanism when considering FLD as a T2DM risk factor. Emerging evidence supports the genetic determinants of NAFLD, with some genes such as TM6SF2 influencing IR and T2DM.^[47]^

ABCC2 is an organic anion transporter expressed on the apical membranes of polarized cells, including hepatocytes, renal epithelia, intestinal epithelia, and placenta. Twelve-year clinical research on pharmacogene variants associated with liver transplantation showed that variants in different locations of the ABCC2 transporter genes were associated with a lower risk of T2DM.^[48]^ A review of 485 participants, including 10 cross-sectional studies, revealed significant changes in liver transporters during the progression of NAFLD to NASH compared with the control group. NAFLD progression is associated with reduced expression and function of several hepatic uptake transporters, upregulation of many efflux transporters, downregulation of cholesterol efflux transporters, and mislocalization of the canalicular transporter ABCC2.^[49]^

This study, combined with existing knowledge on NAFLD and T2DM mechanisms and related research, reveals essential pathways affecting NAFLD and T2DM, including the regulation of developmental growth, small-molecule metabolism, cellular processes, and TG metabolism. These pathways are involved in fat cell differentiation, membrane organization, and cellular respiration. Bioinformatics prediction and experimental verification of key biomarkers for diabetic kidney disease, based on transcriptome sequencing in mice and GO analysis, showed that biological processes are mainly enriched in response to stilbenoids, fatty acids, nutrients, macrophage-derived foam cell differentiation, and TG metabolism.^[50]^ These biological processes influence tissue lipid levels and affect hepatic fat accumulation and IR. Further research is required on this aspect. In conclusion, determining TG, free fatty acid, and cholesterol levels can aid in the clinical observation of dynamic changes in liver fat levels and IR, significantly contributing to comorbidity prediction. As genetic research continues, the development of targeted drugs that regulate liver fat levels and lipid content is anticipated to become a key and effective treatment approach for comorbidities. Once the relevant mechanism of action is identified, specific gene therapies for NAFLD and T2DM are expected to be developed.

NAFLD is strongly linked to high-fat diet (HFD) consumption, which poses a metabolic risk. Although the effects of HFD have been well studied, their impact on the brain at the molecular level remains unclear. Our study, using tissue-specific MAGMA analysis, found significant enrichment of NAFLD in multiple brain tissues, a finding that has not been widely reported. However, evidence supports this finding. A zebrafish study showed that long-term HFD exposure led to increased pro-inflammatory, apoptotic, and proliferative markers in liver and brain tissues.^[50]^ This passage indirectly suggests the potential impact of brain tissue on the development of NAFLD and its possible association with the concurrent occurrence of T2DM. Melatonin (MEL), a hormone produced by the pineal gland that possesses antioxidant and anti-inflammatory properties, has been implicated in circadian rhythm-related diabetes. Reduced MEL levels and their relationship with insulin are believed to contribute to the development of T2DM.^[51]^ Another study has demonstrated that MEL regulates neurodegenerative complications associated with NAFLD by enhancing neurotransmission and maintaining cellular integrity. MEL reduces monoamine turnover and increases brain 8-OHdG levels. It also counteracts elevated levels of GSH, NOx, MDA, and TNF-α in the liver and brain tissues. Therefore, MEL is considered a promising candidate for the treatment of the neurological side effects associated with NAFLD.

In this genome-wide pleiotropic association study, we found extensive genome-wide genetic correlations and overlap between T2DM and NAFLD. Further comprehensive analyses highlighted the pleiotropic genetic variants and loci, potential shared causal variants, pleiotropic genes, biological pathways, and potential genetic basis associated with comorbidities. This study is not the first to reveal the causal association between T2DM and NAFLD, but it had the following innovations: (i) the GWAS database of T2DM used in this study was large and a new GWAS database, which contained a larger population; (ii) it provided strong evidence of genetic correlation and genetic overlap betweenT2DM and NAFLD, and we found that pleiotropic genetic variants, loci, and genes were extensively distributed across the genome, with higher phenotype and tissue specificity.

A limitation of this study was that the shared genes were solely screened from GWAS results in European populations, thereby neglecting other populations. Limited replicated validation studies exist for the susceptibility loci associated with NAFLD and T2DM in other populations. Thus, it remains unclear whether the susceptibility genes identified in this study are present in other populations. These findings may serve as a reference for NAFLD-T2DM research in other populations. GWAS studies should consider diverse populations, sexes, and ages to further explore this wealth of data.

## 5. Conclusion

By integrating GWAS summary data and conditional pleiotropic analysis statistical methods, we identified selective pleiotropy and novel loci shared between T2DM and NAFLD. By combining eQTL analysis, we identified the shared risk genes for T2DM and NAFLD. The MR analysis suggested a nominal causal association between T2DM and NAFLD. These findings could provide novel insights into the genetic overlap between T2DM and NAFLD and help better understand the etiology of both diseases. They not only support the shared genetic basis underlying these diseases but also have important implications for intervention and treatment targets.

## Author contributions

**Formal analysis:** Yao Lu.

**Methodology:** Wenjuan Ni.

**Project administration:** Wei Wang.

**Resources:** Wei Wang.

**Supervision:** Wei Wang.

**Writing – original draft:** Wenjuan Ni.

**Writing – review & editing:** Wei Wang.

## Supplementary Material












































