# Supplementary material for: Exploring the interconnected between type 2 diabetes mellitus and nonalcoholic fatty liver disease: Genetic correlation and Mendelian randomization analysis
Source: Medicine (Baltimore). 2024 May 10;103(19):e38008. doi: 10.1097/MD.0000000000038008 (PMC11081543; doi:10.1097/MD.0000000000038008)
Supplement: Supplementary file 1 [file medi-103-e38008-s001.docx]

Table S1 LDSC

|  | p1 | p2 | rg | se | z | p | h2_obs | h2_obs_se | h2_int | h2_int_se | gcov_int | gcov_int_se |
| --- | --- | --- | --- | --- | --- | --- | --- | --- | --- | --- | --- | --- |
| nointercept | NAFLD | T2D | 0.5354 | 0.0547 | 9.7963 | 1.17E-22 | 0.0452 | 0.0019 | 1 | NA | 0 | NA |
| withintercept | NAFLD | T3D | 0.7266 | 0.1529 | 4.7521 | 2.01E-06 | 0.0458 | 0.0022 | 0.9924 | 0.0239 | 0.001 | 0.0059 |
