# Supplementary figures and images for: Exploring the interconnected between type 2 diabetes mellitus and nonalcoholic fatty liver disease: Genetic correlation and Mendelian randomization analysis
Source: Medicine (Baltimore). 2024 May 10;103(19):e38008. doi: 10.1097/MD.0000000000038008 (PMC11081543; doi:10.1097/MD.0000000000038008)

Figure S1 QQ plot for pleiotropic analysis


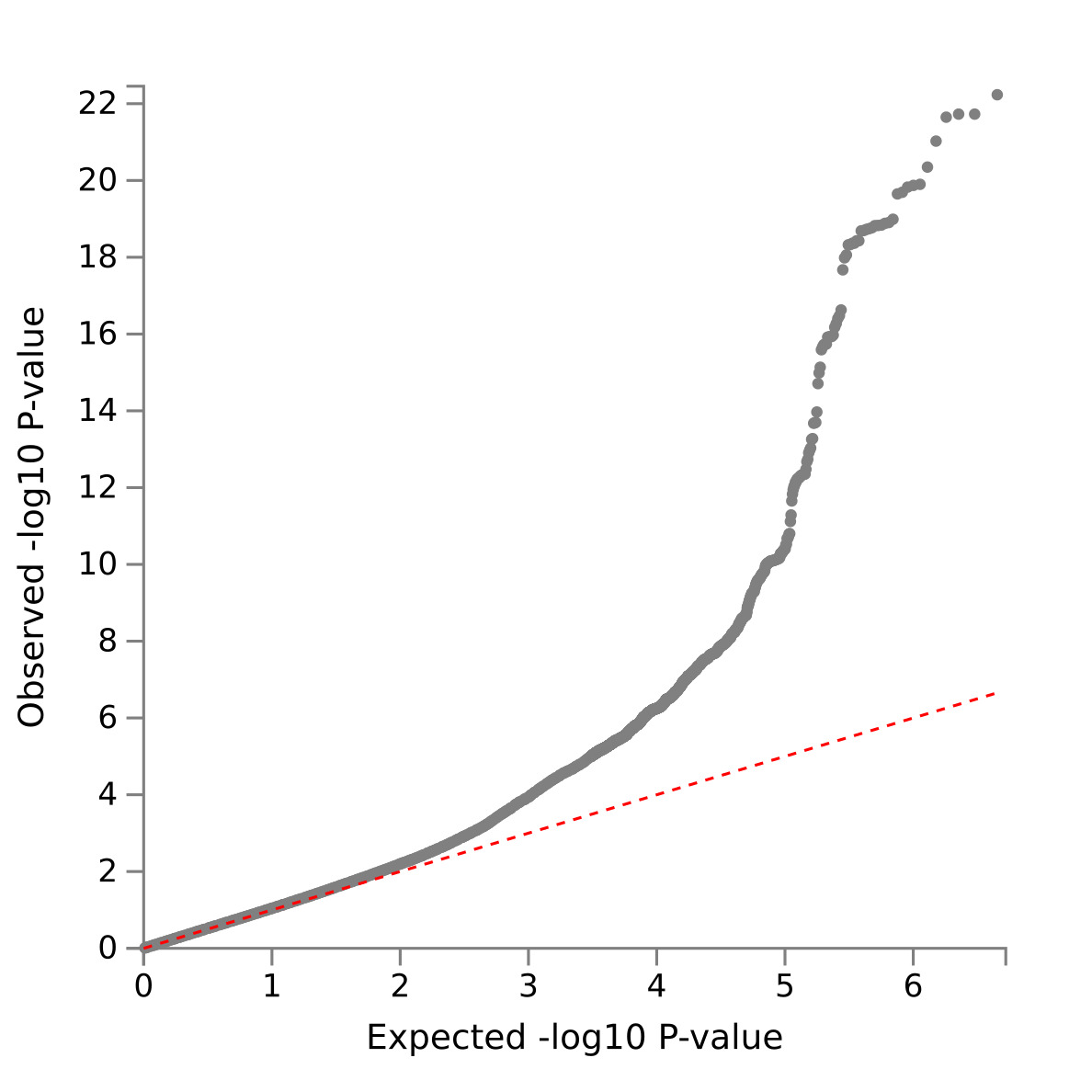

Supplement: Supplementary file 2 [file medi-103-e38008-s002.docx]

Figure S3 Effect of pleiotropic SNP on gene function


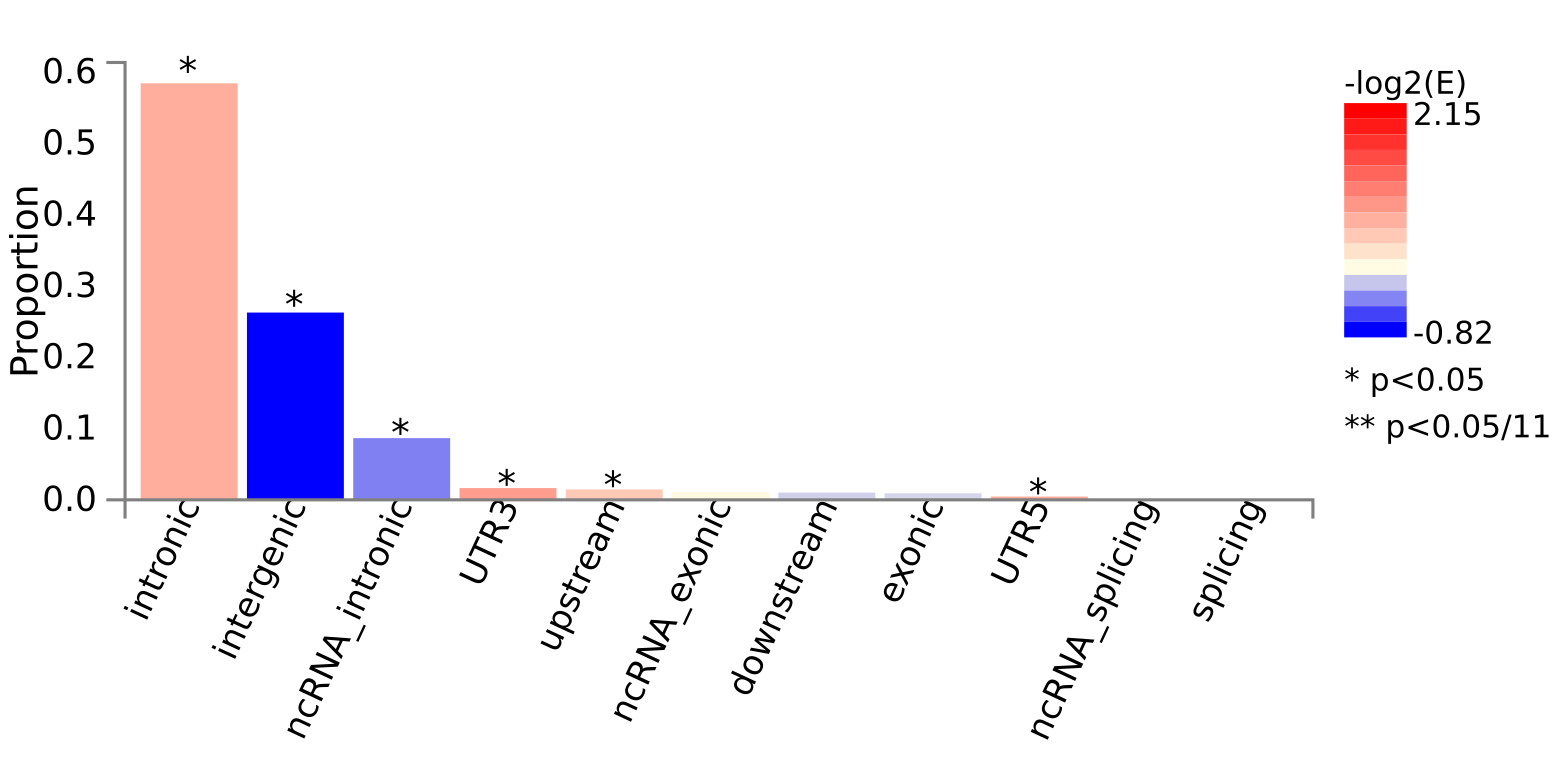

Supplement: Supplementary file 4 [file medi-103-e38008-s004.docx]

Figure S4 Tissue specificity analysis of MAGMA based on genome-wide pleiotropy


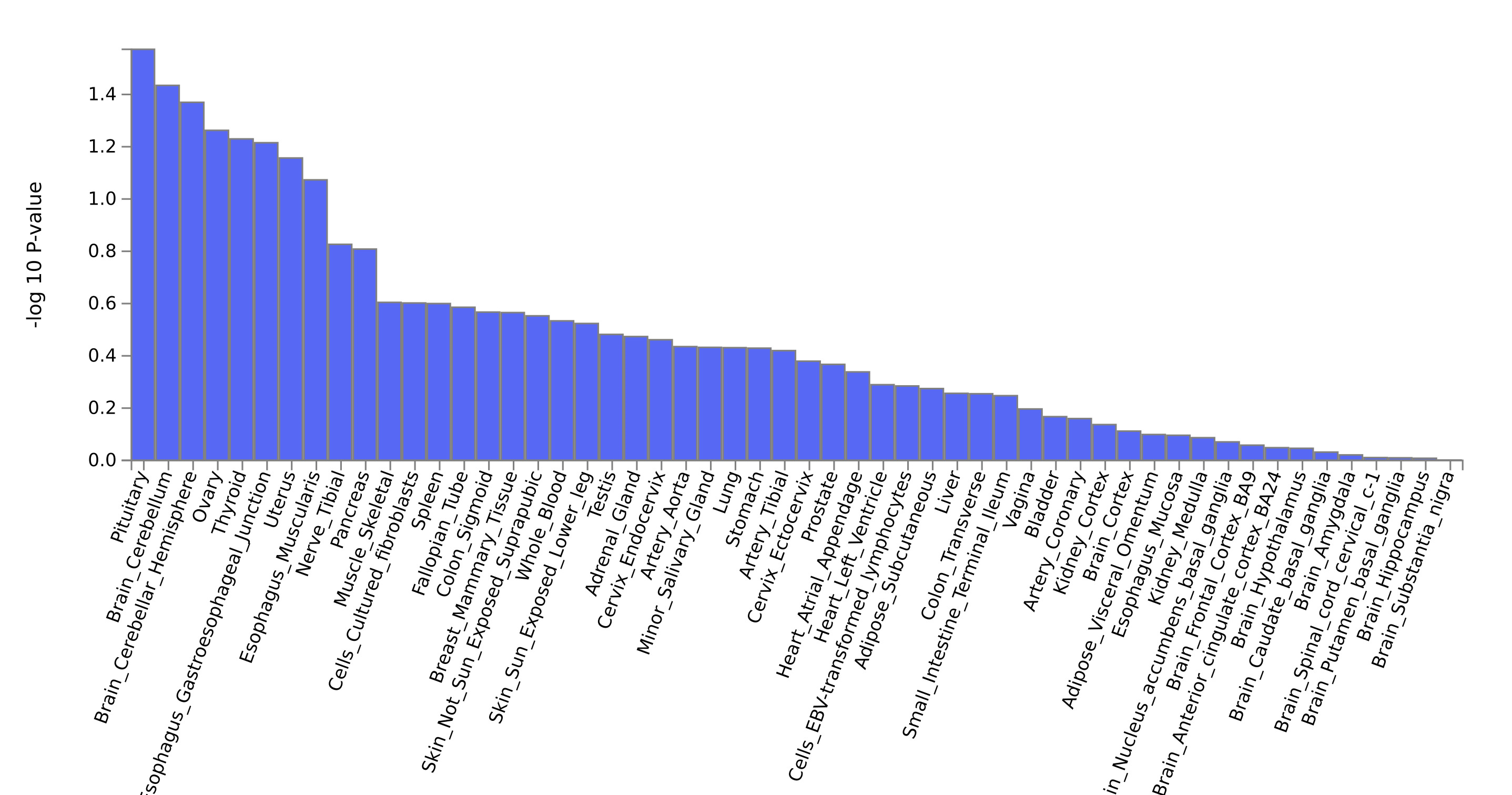

Supplement: Supplementary file 5 [file medi-103-e38008-s005.docx]

Figure S5 Heat map of MAGMA gene analysis


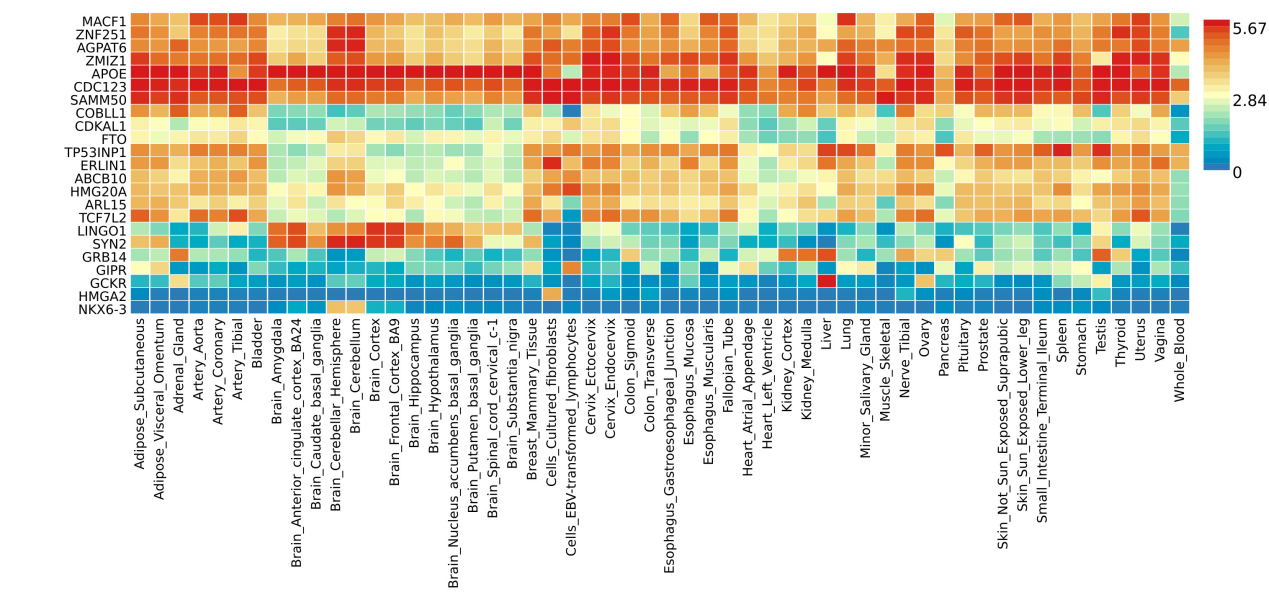

Supplement: Supplementary file 7 [file medi-103-e38008-s007.docx]

Figure S6 Expression of pleiotropic genes in different tissues


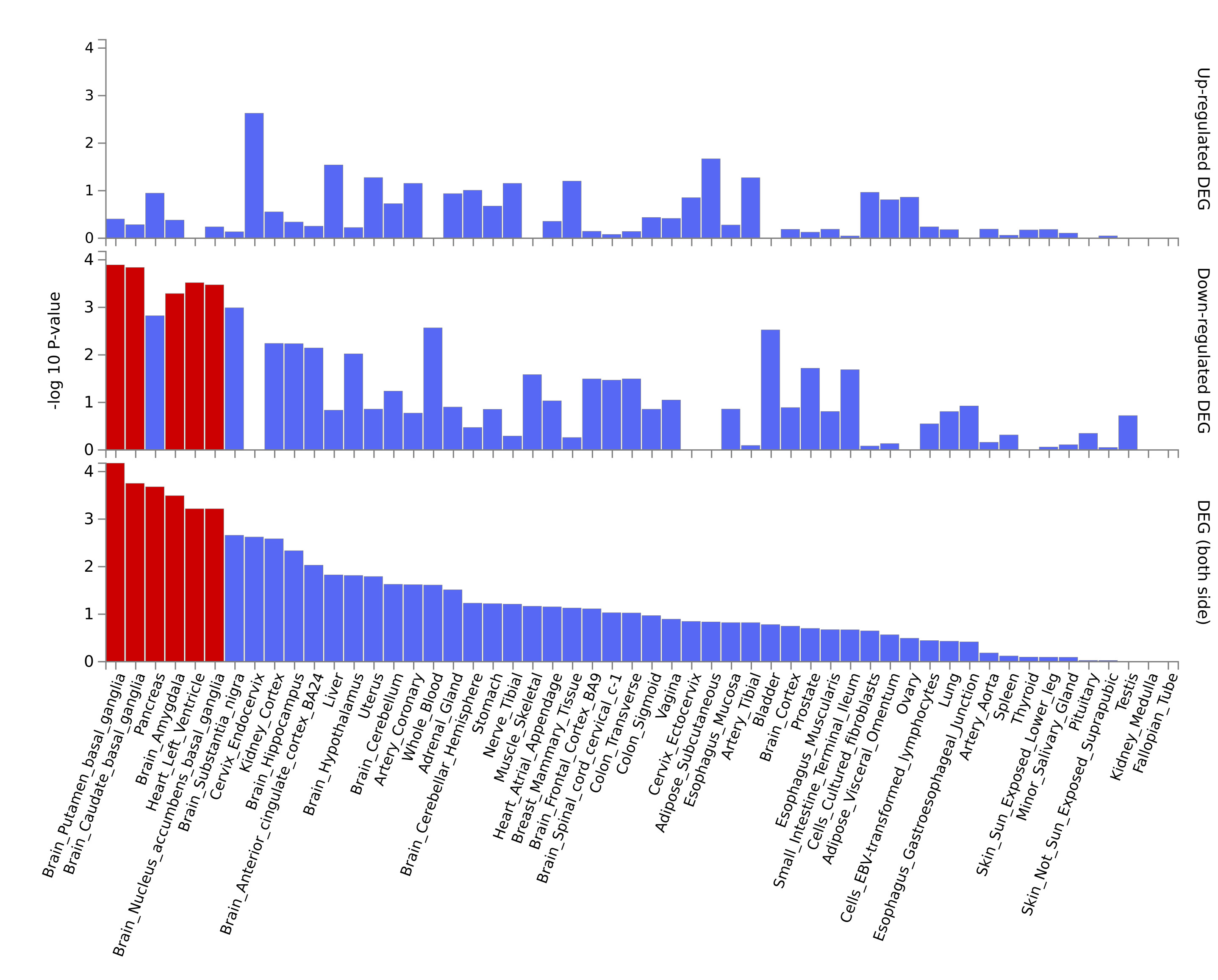

Supplement: Supplementary file 8 [file medi-103-e38008-s008.docx]

Figure S9 Manhattan map of pleiotropic gene between T2D and NAFLD (based on MAGMA gene test).


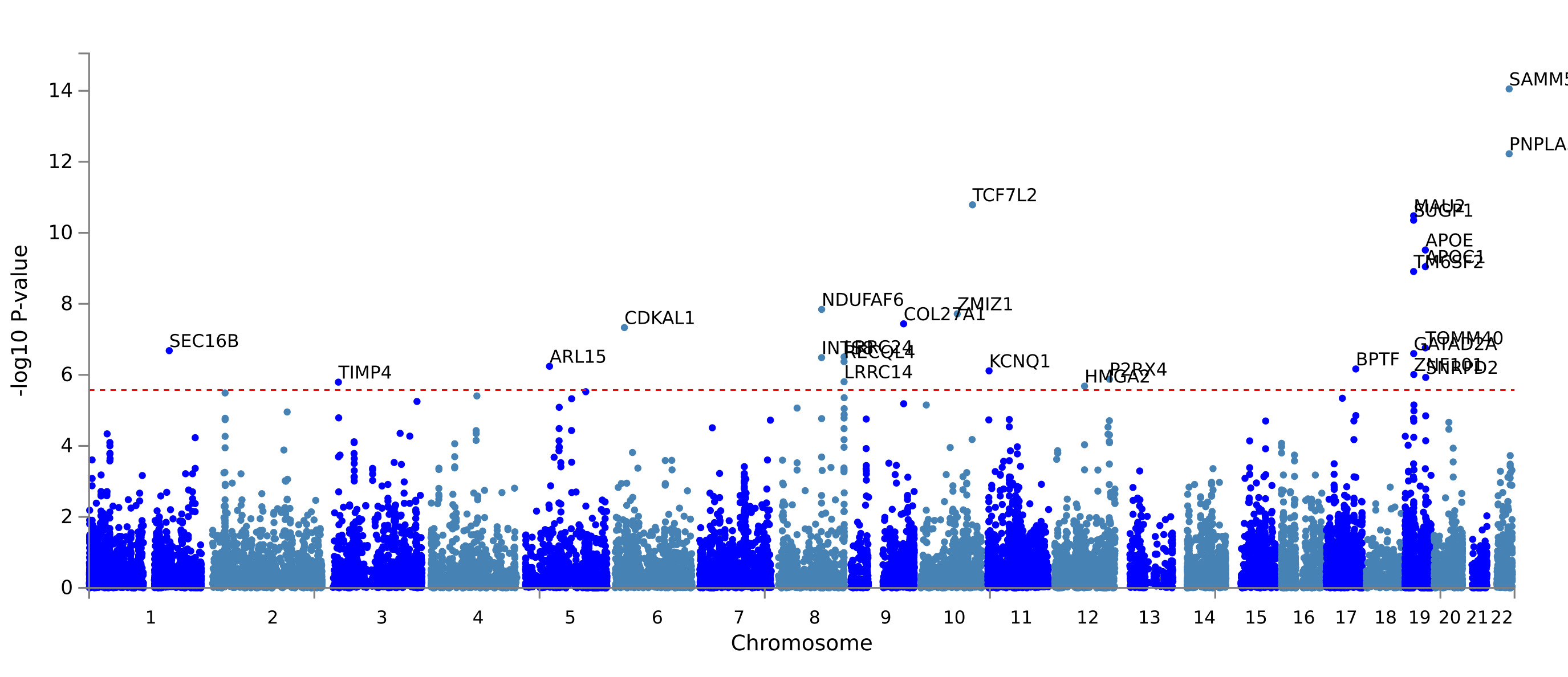

Supplement: Supplementary file 12 [file medi-103-e38008-s012.docx]

Figure S10 Heat map of MAGMA gene analysis


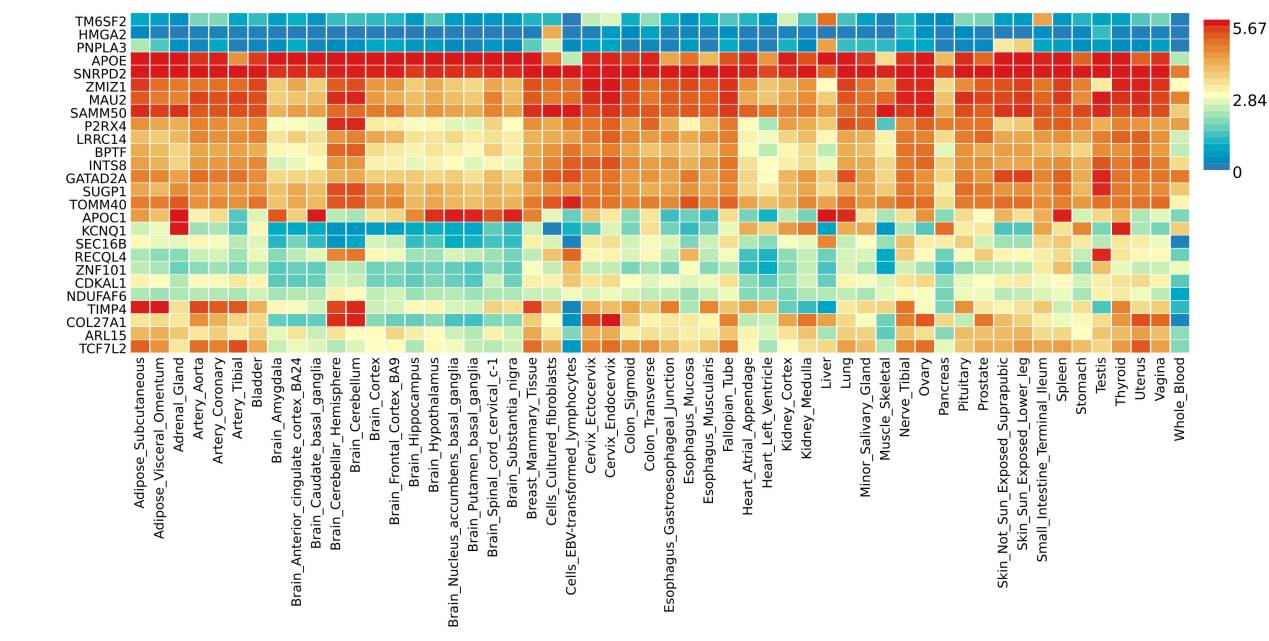

Supplement: Supplementary file 13 [file medi-103-e38008-s013.docx]

Figure S11 The pathway enrichment of pleiotropic genes (KEGG, wiki, GO) based on Magma gene-test


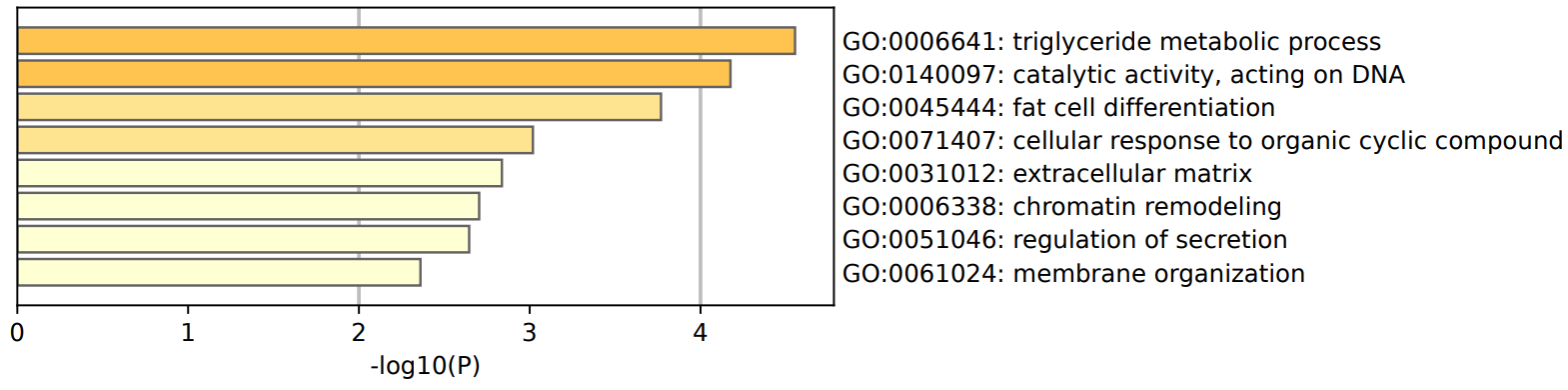

Supplement: Supplementary file 14 [file medi-103-e38008-s014.docx]

Figure S12 Cell-type enrichment of pleiotropic genes based on Magma gene-test


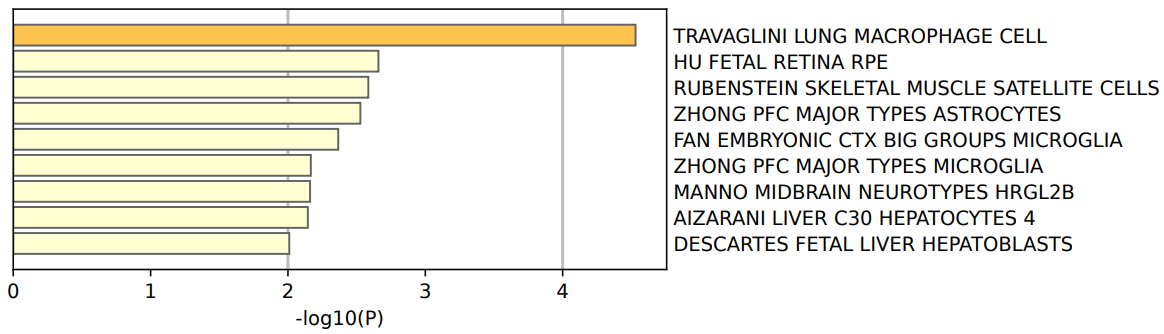

Supplement: Supplementary file 15 [file medi-103-e38008-s015.docx]

Figure S13 Heat map of MAGMA gene analysis


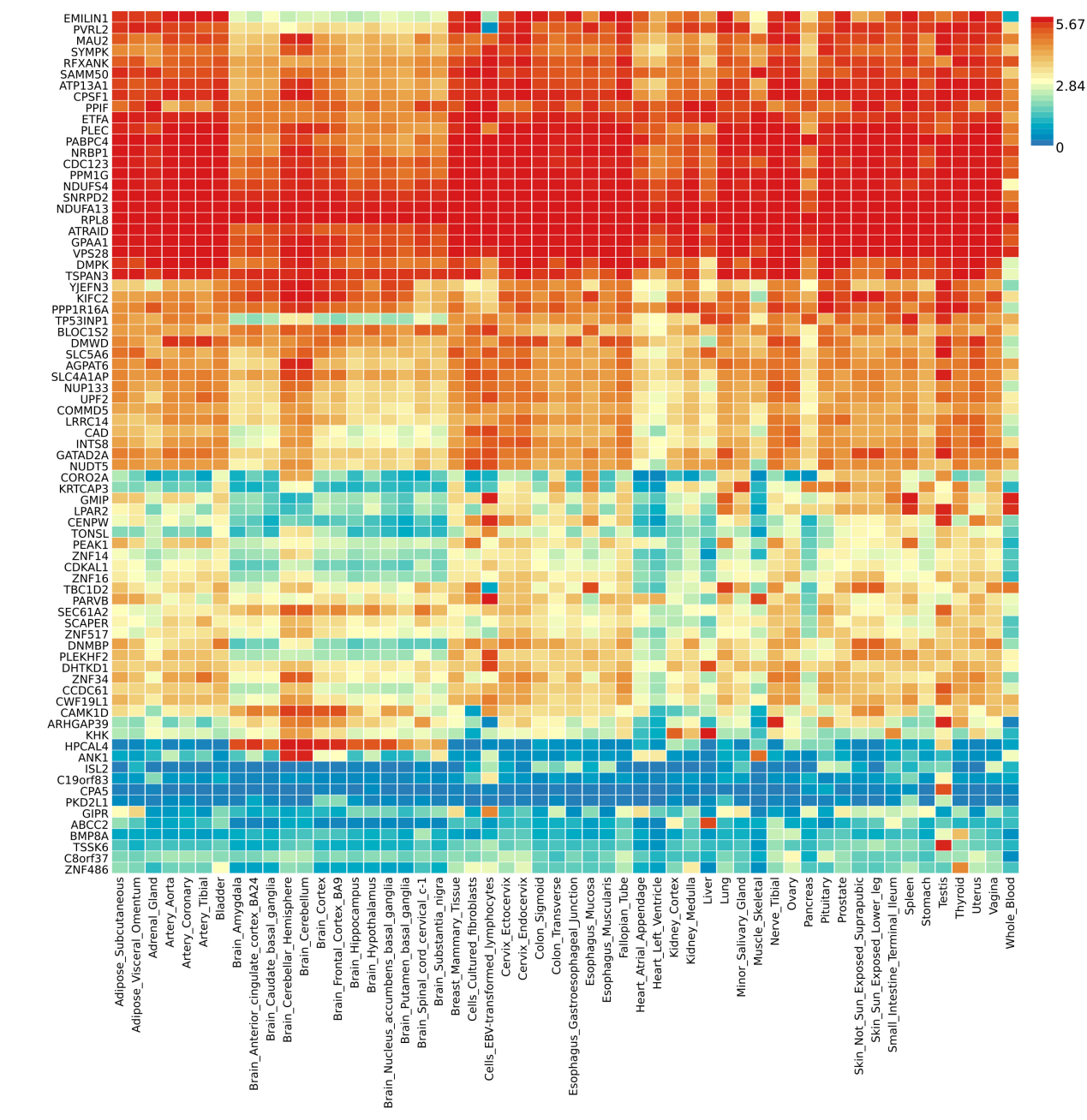

Supplement: Supplementary file 18 [file medi-103-e38008-s018.docx]

Figure S14 The expression of pleiotropic gene in different tissues


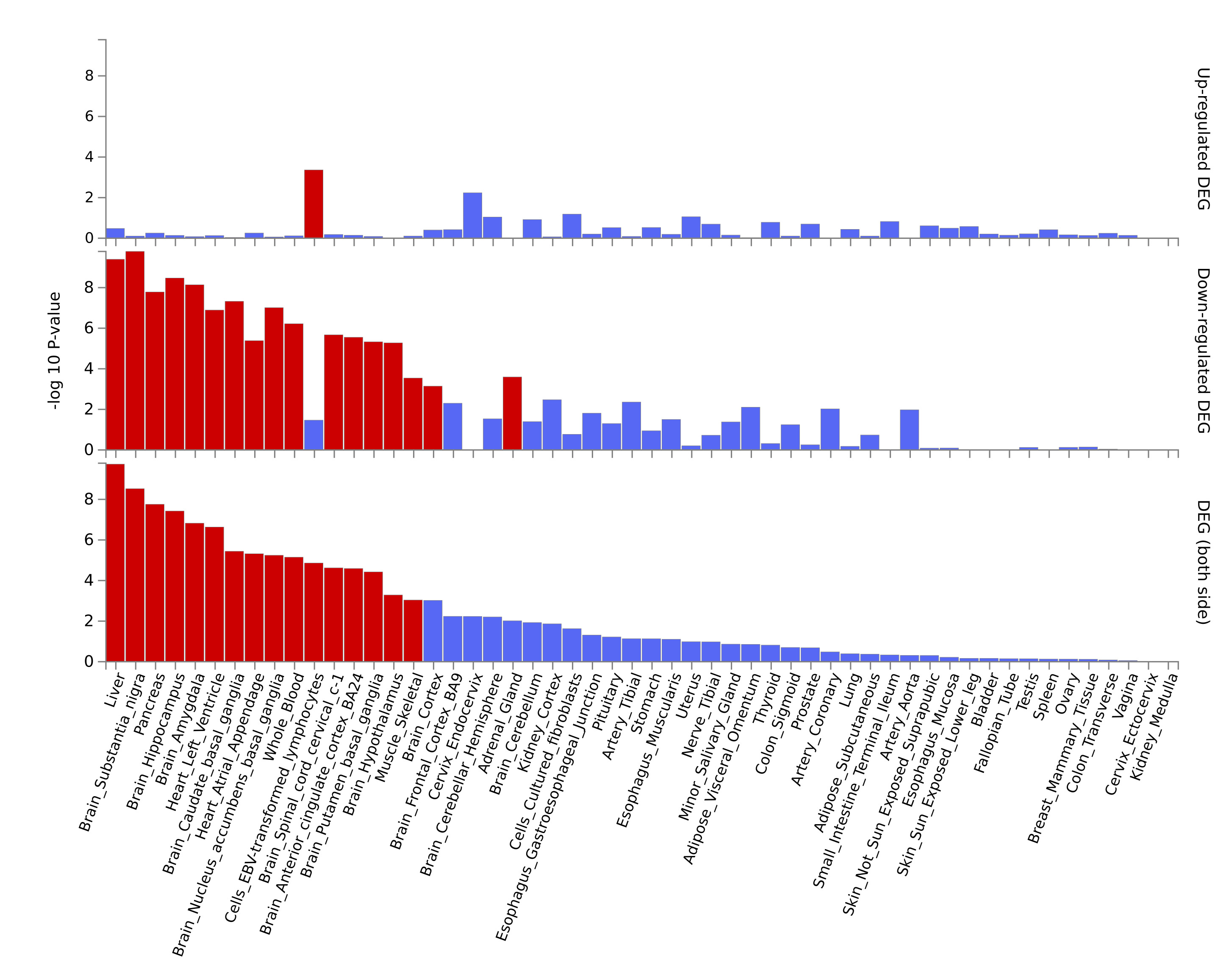

Supplement: Supplementary file 19 [file medi-103-e38008-s019.docx]

Figure S15 The pathway enrichment of pleiotropic genes (KEGG, wiki, GO) based on eQTL analysis


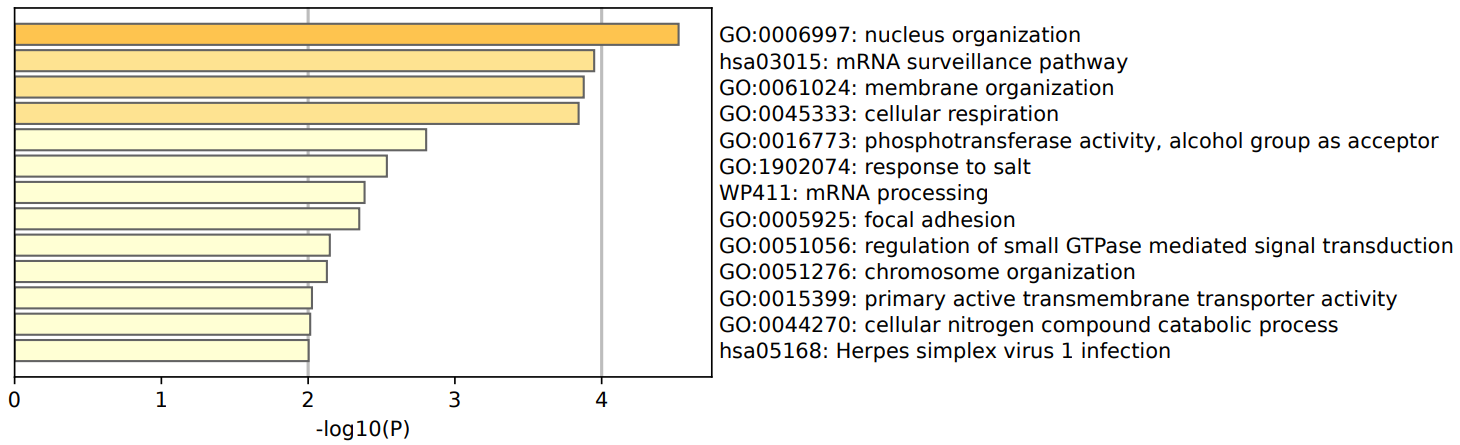

Supplement: Supplementary file 20 [file medi-103-e38008-s020.docx]

Figure S16 The cell-type enrichment of pleiotropic genes based on eQTL analysis


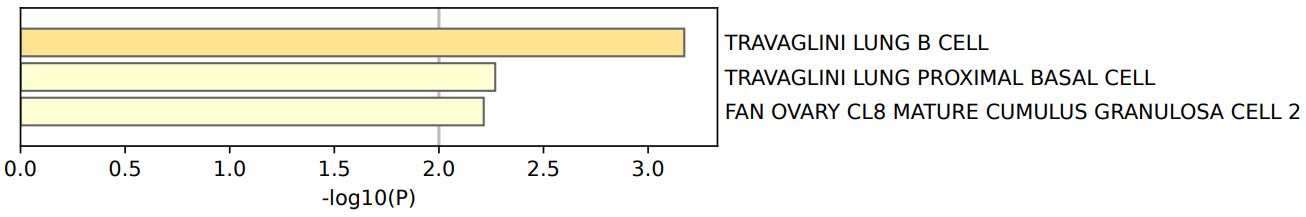

Supplement: Supplementary file 21 [file medi-103-e38008-s021.docx]
