# Supplementary material for: Exploring the interconnected between type 2 diabetes mellitus and nonalcoholic fatty liver disease: Genetic correlation and Mendelian randomization analysis
Source: Medicine (Baltimore). 2024 May 10;103(19):e38008. doi: 10.1097/MD.0000000000038008 (PMC11081543; doi:10.1097/MD.0000000000038008)

Figure S2 Summary of basic information of each genome risk locus (from left to right, the size of risk locus, the number of SNP, the number of Map genes and the number of genes located in the locus).


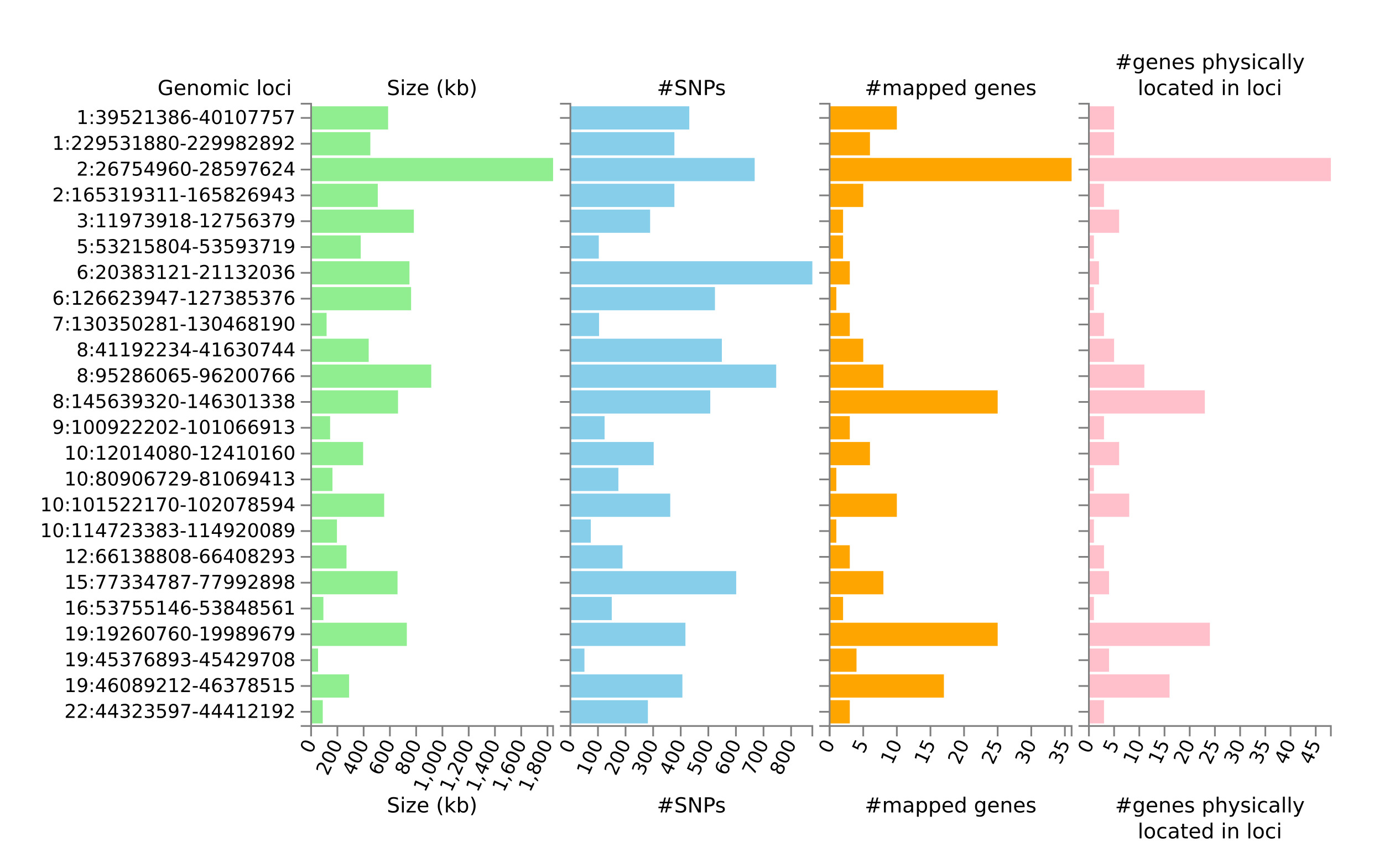

Supplement: Supplementary file 3 [file medi-103-e38008-s003.docx]
