# Supplementary material for: Exploring the interconnected between type 2 diabetes mellitus and nonalcoholic fatty liver disease: Genetic correlation and Mendelian randomization analysis
Source: Medicine (Baltimore). 2024 May 10;103(19):e38008. doi: 10.1097/MD.0000000000038008 (PMC11081543; doi:10.1097/MD.0000000000038008)
Supplement: Supplementary file 6 [file medi-103-e38008-s006.docx]

Table S2 Tissue-specific

| **VARIABLE** | **TYPE** | **NGENES** | **BETA** | **BETA_STD** | **SE** | **P** |
| --- | --- | --- | --- | --- | --- | --- |
| Pituitary | COVAR | 17055 | 0.018 | 0.034 | 0.010 | 0.027 |
| Brain_Cerebellum | COVAR | 17055 | 0.012 | 0.024 | 0.007 | 0.037 |
| Brain_Cerebellar_Hemisphere | COVAR | 17055 | 0.011 | 0.022 | 0.006 | 0.043 |
| Ovary | COVAR | 17055 | 0.017 | 0.034 | 0.010 | 0.055 |
| Thyroid | COVAR | 17055 | 0.016 | 0.032 | 0.010 | 0.059 |
| Esophagus_Gastroesophageal_Junction | COVAR | 17055 | 0.021 | 0.042 | 0.014 | 0.061 |
| Uterus | COVAR | 17055 | 0.017 | 0.035 | 0.012 | 0.070 |
| Esophagus_Muscularis | COVAR | 17055 | 0.018 | 0.036 | 0.013 | 0.085 |
| Nerve_Tibial | COVAR | 17055 | 0.011 | 0.022 | 0.011 | 0.149 |
| Pancreas | COVAR | 17055 | 0.009 | 0.015 | 0.009 | 0.155 |
| Muscle_Skeletal | COVAR | 17055 | 0.005 | 0.010 | 0.007 | 0.249 |
| Cells_Cultured_fibroblasts | COVAR | 17055 | 0.005 | 0.010 | 0.007 | 0.250 |
| Spleen | COVAR | 17055 | 0.005 | 0.010 | 0.008 | 0.251 |
| Fallopian_Tube | COVAR | 17055 | 0.008 | 0.016 | 0.013 | 0.260 |
| Colon_Sigmoid | COVAR | 17055 | 0.008 | 0.016 | 0.013 | 0.271 |
| Breast_Mammary_Tissue | COVAR | 17055 | 0.008 | 0.016 | 0.014 | 0.272 |
| Skin_Not_Sun_Exposed_Suprapubic | COVAR | 17055 | 0.005 | 0.009 | 0.008 | 0.280 |
| Whole_Blood | COVAR | 17055 | 0.003 | 0.006 | 0.006 | 0.293 |
| Skin_Sun_Exposed_Lower_leg | COVAR | 17055 | 0.004 | 0.008 | 0.008 | 0.299 |
| Testis | COVAR | 17055 | 0.003 | 0.005 | 0.006 | 0.330 |
| Adrenal_Gland | COVAR | 17055 | 0.005 | 0.009 | 0.011 | 0.336 |
| Cervix_Endocervix | COVAR | 17055 | 0.005 | 0.010 | 0.012 | 0.346 |
| Artery_Aorta | COVAR | 17055 | 0.004 | 0.008 | 0.011 | 0.367 |
| Minor_Salivary_Gland | COVAR | 17055 | 0.003 | 0.006 | 0.010 | 0.370 |
| Lung | COVAR | 17055 | 0.003 | 0.006 | 0.010 | 0.371 |
| Stomach | COVAR | 17055 | 0.004 | 0.007 | 0.013 | 0.372 |
| Artery_Tibial | COVAR | 17055 | 0.003 | 0.007 | 0.011 | 0.380 |
| Cervix_Ectocervix | COVAR | 17055 | 0.003 | 0.005 | 0.013 | 0.417 |
| Prostate | COVAR | 17055 | 0.002 | 0.004 | 0.013 | 0.429 |
| Heart_Atrial_Appendage | COVAR | 17055 | 0.001 | 0.002 | 0.010 | 0.459 |
| Heart_Left_Ventricle | COVAR | 17055 | 0.000 | -0.001 | 0.010 | 0.513 |
| Cells_EBV-transformed_lymphocytes | COVAR | 17055 | 0.000 | -0.001 | 0.005 | 0.519 |
| Adipose_Subcutaneous | COVAR | 17055 | -0.001 | -0.002 | 0.012 | 0.531 |
| Liver | COVAR | 17055 | -0.001 | -0.002 | 0.007 | 0.554 |
| Colon_Transverse | COVAR | 17055 | -0.002 | -0.003 | 0.012 | 0.556 |
| Small_Intestine_Terminal_Ileum | COVAR | 17055 | -0.002 | -0.003 | 0.010 | 0.566 |
| Vagina | COVAR | 17055 | -0.004 | -0.008 | 0.012 | 0.636 |
| Bladder | COVAR | 17055 | -0.007 | -0.013 | 0.014 | 0.680 |
| Artery_Coronary | COVAR | 17055 | -0.006 | -0.013 | 0.013 | 0.692 |
| Kidney_Cortex | COVAR | 17055 | -0.006 | -0.010 | 0.010 | 0.730 |
| Brain_Cortex | COVAR | 17055 | -0.006 | -0.010 | 0.008 | 0.773 |
| Adipose_Visceral_Omentum | COVAR | 17055 | -0.010 | -0.019 | 0.012 | 0.796 |
| Esophagus_Mucosa | COVAR | 17055 | -0.007 | -0.013 | 0.008 | 0.802 |
| Kidney_Medulla | COVAR | 17055 | -0.009 | -0.017 | 0.010 | 0.819 |
| Brain_Nucleus_accumbens_basal_ganglia | COVAR | 17055 | -0.008 | -0.014 | 0.008 | 0.850 |
| Brain_Frontal_Cortex_BA9 | COVAR | 17055 | -0.008 | -0.015 | 0.007 | 0.875 |
| Brain_Anterior_cingulate_cortex_BA24 | COVAR | 17055 | -0.010 | -0.017 | 0.008 | 0.894 |
| Brain_Hypothalamus | COVAR | 17055 | -0.011 | -0.019 | 0.009 | 0.899 |
| Brain_Caudate_basal_ganglia | COVAR | 17055 | -0.012 | -0.021 | 0.008 | 0.930 |
| Brain_Amygdala | COVAR | 17055 | -0.014 | -0.024 | 0.008 | 0.953 |
| Brain_Spinal_cord_cervical_c-1 | COVAR | 17055 | -0.018 | -0.032 | 0.009 | 0.976 |
| Brain_Putamen_basal_ganglia | COVAR | 17055 | -0.017 | -0.029 | 0.008 | 0.978 |
| Brain_Hippocampus | COVAR | 17055 | -0.018 | -0.030 | 0.008 | 0.982 |
| Brain_Substantia_nigra | COVAR | 17055 | -0.025 | -0.043 | 0.009 | 0.997 |
