# Supplementary material for: Exploring the interconnected between type 2 diabetes mellitus and nonalcoholic fatty liver disease: Genetic correlation and Mendelian randomization analysis
Source: Medicine (Baltimore). 2024 May 10;103(19):e38008. doi: 10.1097/MD.0000000000038008 (PMC11081543; doi:10.1097/MD.0000000000038008)

Figure S8 The cell-type enrichment of pleiotropic genes through the position information of lead SNP


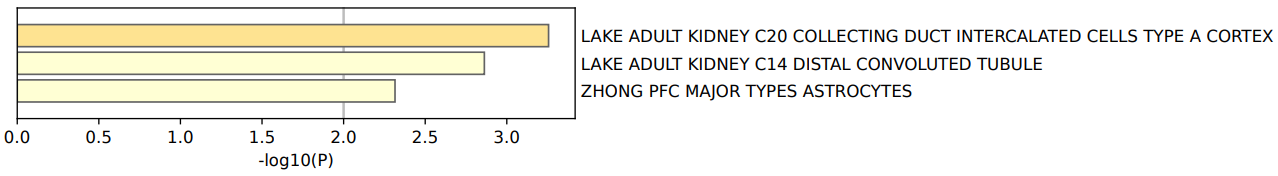

Supplement: Supplementary file 10 [file medi-103-e38008-s010.docx]
