# Supplementary material for: Exploring the interconnected between type 2 diabetes mellitus and nonalcoholic fatty liver disease: Genetic correlation and Mendelian randomization analysis
Source: Medicine (Baltimore). 2024 May 10;103(19):e38008. doi: 10.1097/MD.0000000000038008 (PMC11081543; doi:10.1097/MD.0000000000038008)
Supplement: Supplementary file 11 [file medi-103-e38008-s011.docx]

Table S3 Magma_gene

| **GENE** | **CHR** | **START** | **STOP** | **NSNPS** | **NPARAM** | **N** | **ZSTAT** | **P** | **SYMBOL** |
| --- | --- | --- | --- | --- | --- | --- | --- | --- | --- |
| ENSG00000100347 | 22 | 44351301 | 44406411 | 188 | 28 | 655623 | 7.6659 | 8.88E-15 | SAMM50 |
| ENSG00000100344 | 22 | 44319619 | 44360368 | 109 | 20 | 655623 | 7.1061 | 5.97E-13 | PNPLA3 |
| ENSG00000148737 | 10 | 114710009 | 114927437 | 461 | 95 | 655623 | 6.6349 | 1.62E-11 | TCF7L2 |
| ENSG00000129933 | 19 | 19431490 | 19469563 | 104 | 17 | 655623 | 6.5291 | 3.31E-11 | MAU2 |
| ENSG00000105705 | 19 | 19386827 | 19431653 | 114 | 19 | 655623 | 6.4857 | 4.42E-11 | SUGP1 |
| ENSG00000130203 | 19 | 45409011 | 45412650 | 6 | 3 | 655623 | 6.1877 | 3.05E-10 | APOE |
| ENSG00000130208 | 19 | 45417504 | 45422606 | 10 | 5 | 655623 | 6.015 | 8.99E-10 | APOC1 |
| ENSG00000213996 | 19 | 19375173 | 19384200 | 27 | 8 | 655623 | 5.964 | 1.23E-09 | TM6SF2 |
| ENSG00000156170 | 8 | 95907995 | 96128683 | 677 | 40 | 655623 | 5.5489 | 1.44E-08 | NDUFAF6 |
| ENSG00000108175 | 10 | 80828792 | 81076276 | 937 | 140 | 655623 | 5.4988 | 1.91E-08 | ZMIZ1 |
| ENSG00000196739 | 9 | 116917840 | 117074791 | 662 | 101 | 655623 | 5.3833 | 3.66E-08 | COL27A1 |
| ENSG00000145996 | 6 | 20534688 | 21232635 | 2142 | 102 | 655623 | 5.3396 | 4.66E-08 | CDKAL1 |
| ENSG00000130204 | 19 | 45393826 | 45406946 | 58 | 14 | 655623 | 5.0958 | 1.74E-07 | TOMM40 |
| ENSG00000120341 | 1 | 177893091 | 177953438 | 247 | 44 | 655623 | 5.0613 | 2.08E-07 | SEC16B |
| ENSG00000167491 | 19 | 19496635 | 19619740 | 365 | 25 | 655623 | 5.0249 | 2.52E-07 | GATAD2A |
| ENSG00000254402 | 8 | 145747761 | 145752416 | 9 | 5 | 655623 | 4.9839 | 3.12E-07 | LRRC24 |
| ENSG00000164941 | 8 | 95825539 | 95893974 | 213 | 22 | 655623 | 4.9745 | 3.27E-07 | INTS8 |
| ENSG00000160957 | 8 | 145736667 | 145743229 | 24 | 7 | 655623 | 4.9241 | 4.24E-07 | RECQL4 |
| ENSG00000185305 | 5 | 53179775 | 53606412 | 1541 | 98 | 655623 | 4.8646 | 5.73E-07 | ARL15 |
| ENSG00000171634 | 17 | 65821640 | 65980494 | 282 | 18 | 655623 | 4.8302 | 6.82E-07 | BPTF |
| ENSG00000053918 | 11 | 2465914 | 2870339 | 1482 | 167 | 655623 | 4.8059 | 7.70E-07 | KCNQ1 |
| ENSG00000181896 | 19 | 19779605 | 19794318 | 39 | 13 | 655623 | 4.7581 | 9.77E-07 | ZNF101 |
| ENSG00000125743 | 19 | 46190712 | 46195827 | 10 | 4 | 655623 | 4.7201 | 1.18E-06 | SNRPD2 |
| ENSG00000135124 | 12 | 121647660 | 121671909 | 97 | 15 | 655623 | 4.6964 | 1.32E-06 | P2RX4 |
| ENSG00000160959 | 8 | 145743376 | 145750557 | 18 | 8 | 655623 | 4.662 | 1.57E-06 | LRRC14 |
| ENSG00000157150 | 3 | 12194551 | 12200851 | 20 | 7 | 655623 | 4.6569 | 1.61E-06 | TIMP4 |
| ENSG00000149948 | 12 | 66217911 | 66360075 | 278 | 46 | 655623 | 4.6033 | 2.08E-06 | HMGA2 |
