# Supplementary material for: Exploring the interconnected between type 2 diabetes mellitus and nonalcoholic fatty liver disease: Genetic correlation and Mendelian randomization analysis
Source: Medicine (Baltimore). 2024 May 10;103(19):e38008. doi: 10.1097/MD.0000000000038008 (PMC11081543; doi:10.1097/MD.0000000000038008)
Supplement: Supplementary file 17 [file medi-103-e38008-s017.docx]

Table S5 eQTL_gene

| **ENSG** | **entrezID** | **symbol** | **hgnc_symbol** | **OMIM** | **uniprotID** | **DrugBank** |
| --- | --- | --- | --- | --- | --- | --- |
| ENSG00000183682 | 353500 | BMP8A | BMP8A | NA | Q7Z5Y6 | NA |
| ENSG00000090621 | 8761 | PABPC4 | PABPC4 | 603407 | Q13310 | NA |
| ENSG00000116983 | 51440 | HPCAL4 | HPCAL4 | NA | Q9UM19 | NA |
| ENSG00000069248 | 55746 | NUP133 | NUP133 | 607613 | Q8WUM0 | NA |
| ENSG00000151461 | 26019 | UPF2 | UPF2 | 605529 | Q9HAU5 | NA |
| ENSG00000181192 | 55526 | DHTKD1 | DHTKD1 | 614984 | Q96HY7 | NA |
| ENSG00000065665 | 55176 | SEC61A2 | SEC61A2 | NA | Q9H9S3 | NA |
| ENSG00000165609 | 11164 | NUDT5 | NUDT5 | 609230 | Q9UKK9 | NA |
| ENSG00000151465 | 8872 | CDC123 | CDC123 | 617708 | O75794 | NA |
| ENSG00000183049 | 57118 | CAMK1D | CAMK1D | 607957 | Q8IU85 | DB08454:DB12010 |
| ENSG00000108179 | 10105 | PPIF | PPIF | 604486 | P30405 | DB00091:DB00172:DB02078:DB08168 |
| ENSG00000023839 | 1244 | ABCC2 | ABCC2 | 601107 | Q92887 | DB00171:DB01138 |
| ENSG00000107554 | 23268 | DNMBP | DNMBP | 611282 | Q6XZF7 | NA |
| ENSG00000095485 | 55280 | CWF19L1 | CWF19L1 | 616120 | Q69YN2 | NA |
| ENSG00000196072 | 282991 | BLOC1S2 | BLOC1S2 | 609768 | Q6QNY1 | NA |
| ENSG00000107593 | 9033 | PKD2L1 | PKD2L1 | 604532 | Q9P0L9 | NA |
| ENSG00000140374 | 2108 | ETFA | ETFA | 608053 | P13804 | NA |
| ENSG00000159556 | 64843 | ISL2 | ISL2 | 609481 | Q96A47 | NA |
| ENSG00000140386 | 49855 | SCAPER | SCAPER | 611611 | Q9BY12 | NA |
| ENSG00000140391 | 10099 | TSPAN3 | TSPAN3 | 613134 | O60637 | NA |
| ENSG00000173517 | 79834 | PEAK1 | PEAK1 | 614248 | Q9H792 | NA |
| ENSG00000064490 | 8625 | RFXANK | RFXANK | 603200 | O14593 | NA |
| ENSG00000129933 | 23383 | MAU2 | MAU2 | 614560 | Q9Y6X3 | NA |
| ENSG00000167491 | 54815 | GATAD2A | GATAD2A | 614997 | Q86YP4 | NA |
| ENSG00000178093 | 83983 | TSSK6 | TSSK6 | 610712 | Q9BXA6 | NA |
| ENSG00000186010 | 51079 | NDUFA13 | NDUFA13 | 609435 | Q9P0J0 | DB00157 |
| ENSG00000250067 | 374887 | YJEFN3 | YJEFN3 | NA | A6XGL0 | NA |
| ENSG00000064547 | 9170 | LPAR2 | LPAR2 | 605110 | Q9HBW0 | NA |
| ENSG00000089639 | 51291 | GMIP | GMIP | 609694 | Q9P107 | NA |
| ENSG00000105726 | 57130 | ATP13A1 | ATP13A1 | NA | Q9HD20 | NA |
| ENSG00000105708 | 7561 | ZNF14 | ZNF14 | 194556 | P17017 | NA |
| ENSG00000256229 | 90649 | ZNF486 | ZNF486 | NA | Q96H40 | NA |
| ENSG00000130202 | 5819 | PVRL2 | PVRL2 | 600798 | Q92692 | NA |
| ENSG00000267757 | 100287177 | C19orf83 | C19orf83 | NA | NA | NA |
| ENSG00000010310 | 2696 | GIPR | GIPR | 137241 | P48546 | NA |
| ENSG00000125743 | 6633 | SNRPD2 | SNRPD2 | 601061 | P62316 | DB11638 |
| ENSG00000104936 | 1760 | DMPK | DMPK | 605377 | Q09013 | DB01946 |
| ENSG00000185800 | 1762 | DMWD | DMWD | 609857 | Q09019 | NA |
| ENSG00000125755 | 8189 | SYMPK | SYMPK | 602388 | Q92797 | NA |
| ENSG00000104983 | 729440 | CCDC61 | CCDC61 | NA | Q9Y6R9 | NA |
| ENSG00000138080 | 11117 | EMILIN1 | EMILIN1 | 130660 | Q9Y6C2 | NA |
| ENSG00000138030 | 3795 | KHK | KHK | 614058 | P50053 | NA |
| ENSG00000138074 | 8884 | SLC5A6 | SLC5A6 | 604024 | Q9Y289 | DB00121:DB00166 |
| ENSG00000138085 | 51374 | ATRAID | ATRAID | NA | Q6UW56 | NA |
| ENSG00000084774 | 790 | CAD | CAD | 114010 | P27708 | DB00128:DB03459 |
| ENSG00000115241 | 5496 | PPM1G | PPM1G | 605119 | O15355 | NA |
| ENSG00000115216 | 29959 | NRBP1 | NRBP1 | 606010 | Q9UHY1 | NA |
| ENSG00000157992 | 200634 | KRTCAP3 | KRTCAP3 | NA | Q53RY4 | NA |
| ENSG00000163798 | 22950 | SLC4A1AP | SLC4A1AP | 602655 | Q9BWU0 | NA |
| ENSG00000100347 | 25813 | SAMM50 | SAMM50 | 612058 | Q9Y512 | NA |
| ENSG00000188677 | 29780 | PARVB | PARVB | 608121 | Q9HBI1 | NA |
| ENSG00000164258 | 4724 | NDUFS4 | NDUFS4 | 602694 | O43181 | DB00157 |
| ENSG00000145996 | 54901 | CDKAL1 | CDKAL1 | 611259 | Q5VV42 | NA |
| ENSG00000203760 | 387103 | CENPW | CENPW | 611264 | Q5EE01 | NA |
| ENSG00000158525 | 93979 | CPA5 | CPA5 | 609561 | Q8WXQ8 | NA |
| ENSG00000158669 | 137964 | AGPAT6 | AGPAT6 | 608143 | Q86UL3 | NA |
| ENSG00000029534 | 286 | ANK1 | ANK1 | 612641 | P16157 | NA |
| ENSG00000164941 | 55656 | INTS8 | INTS8 | 611351 | Q75QN2 | NA |
| ENSG00000164938 | 94241 | TP53INP1 | TP53INP1 | 606185 | Q96A56 | NA |
| ENSG00000175895 | 79666 | PLEKHF2 | PLEKHF2 | 615208 | Q9H8W4 | NA |
| ENSG00000156172 | 157657 | C8orf37 | C8orf37 | 614477 | Q96NL8 | NA |
| ENSG00000178209 | 5339 | PLEC | PLEC | 601282 | Q15149 | NA |
| ENSG00000197858 | 8733 | GPAA1 | GPAA1 | 603048 | O43292 | NA |
| ENSG00000071894 | 29894 | CPSF1 | CPSF1 | 606027 | Q10570 | NA |
| ENSG00000160948 | 51160 | VPS28 | VPS28 | 611952 | Q9UK41 | NA |
| ENSG00000160949 | 4796 | TONSL | TONSL | 604546 | Q96HA7 | NA |
| ENSG00000167702 | 90990 | KIFC2 | KIFC2 | 615216 | Q96AC6 | NA |
| ENSG00000160972 | 84988 | PPP1R16A | PPP1R16A | 609172 | Q96I34 | NA |
| ENSG00000160959 | 9684 | LRRC14 | LRRC14 | NA | Q15048 | NA |
| ENSG00000147799 | 80728 | ARHGAP39 | ARHGAP39 | 615880 | Q9C0H5 | NA |
| ENSG00000196378 | 80778 | ZNF34 | ZNF34 | 194526 | Q8IZ26 | NA |
| ENSG00000161016 | 6132 | RPL8 | RPL8 | 604177 | P62917 | DB02494:DB07374:DB08437 |
| ENSG00000197363 | 340385 | ZNF517 | ZNF517 | NA | Q6ZMY9 | NA |
| ENSG00000170619 | 28991 | COMMD5 | COMMD5 | 608216 | Q9GZQ3 | NA |
| ENSG00000170631 | 7564 | ZNF16 | ZNF16 | 601262 | P17020 | NA |
| ENSG00000106789 | 7464 | CORO2A | CORO2A | 602159 | Q92828 | NA |
| ENSG00000095383 | 55357 | TBC1D2 | TBC1D2 | 609871 | Q9BYX2 | NA |
