# Supplementary material for: Exploring the interconnected between type 2 diabetes mellitus and nonalcoholic fatty liver disease: Genetic correlation and Mendelian randomization analysis
Source: Medicine (Baltimore). 2024 May 10;103(19):e38008. doi: 10.1097/MD.0000000000038008 (PMC11081543; doi:10.1097/MD.0000000000038008)
Supplement: Supplementary file 22 [file medi-103-e38008-s022.docx]

Table S6 index_SNP

| **Exp** | **SNP** | **CHR** | **POS** | **A1** | **A2** | **BETA** | **SE** | **Z** | **P** | **N** |
| --- | --- | --- | --- | --- | --- | --- | --- | --- | --- | --- |
| NAFLD | rs2745359 | 6 | 127381956 | C | T | 0.301248 | 0.0497304 | 6.057622702 | 1.38E-09 | 377277 |
| NAFLD | rs2954029 | 8 | 126490972 | T | A | 0.176558 | 0.0297947 | -5.925819022 | 3.11E-09 | 377277 |
| NAFLD | rs28703824 | 9 | 100954429 | A | G | 0.234269 | 0.0419815 | 5.580291319 | 2.40E-08 | 377277 |
| NAFLD | rs8100204 | 19 | 19393714 | A | G | 0.329657 | 0.0377815 | 8.725355002 | 2.65E-18 | 377277 |
| NAFLD | rs738409 | 22 | 44324727 | G | C | 0.467268 | 0.0319662 | 14.6175648 | 2.17E-48 | 377277 |
| T2D | rs3768321 | 1 | 40035928 | T | G | 0.084 | 0.008 | 10.5 | 4.75E-26 | 933970 |
| T3D | rs58432198 | 1 | 51256091 | T | C | -0.0636 | 0.0103 | -6.174757282 | 5.71E-10 | 933970 |
| T4D | rs12140153 | 1 | 62579891 | T | G | -0.0645 | 0.0113 | -5.707964602 | 1.17E-08 | 933970 |
| T5D | rs1127215 | 1 | 117532790 | T | C | -0.0491 | 0.0065 | -7.553846154 | 3.92E-14 | 933970 |
| T6D | rs320369 | 1 | 118143517 | A | G | 0.0372 | 0.0068 | 5.470588235 | 4.60E-08 | 933970 |
| T7D | rs1493694 | 1 | 120526982 | T | C | 0.08 | 0.0102 | 7.843137255 | 3.35E-15 | 933970 |
| T8D | rs490689 | 1 | 177873841 | A | G | 0.0543 | 0.008 | 6.7875 | 8.86E-12 | 933970 |
| T9D | rs6687271 | 1 | 205165322 | A | C | -0.0402 | 0.0073 | -5.506849315 | 4.14E-08 | 933970 |
| T10D | rs340874 | 1 | 214159256 | T | C | -0.0678 | 0.0065 | -10.43076923 | 1.56E-25 | 933970 |
| T11D | rs2820441 | 1 | 219734960 | A | C | 0.0556 | 0.0069 | 8.057971014 | 8.56E-16 | 933970 |
| T12D | rs348330 | 1 | 229672955 | A | G | -0.0492 | 0.0067 | -7.343283582 | 2.10E-13 | 933970 |
| T13D | rs62107261 | 2 | 422144 | T | C | 0.1019 | 0.0161 | 6.329192547 | 2.62E-10 | 933970 |
| T14D | rs13022337 | 2 | 632609 | A | G | -0.0519 | 0.0088 | -5.897727273 | 3.61E-09 | 933970 |
| T15D | rs11680058 | 2 | 16574669 | A | G | 0.0581 | 0.0104 | 5.586538462 | 2.08E-08 | 933970 |
| T16D | rs1260326 | 2 | 27730940 | T | C | -0.0644 | 0.0066 | -9.757575758 | 1.62E-22 | 933970 |
| T17D | rs17030845 | 2 | 43687879 | T | C | -0.1191 | 0.0108 | -11.02777778 | 2.35E-28 | 933970 |
| T18D | rs6545714 | 2 | 59307725 | A | G | -0.0363 | 0.0065 | -5.584615385 | 2.25E-08 | 933970 |
| T19D | rs243019 | 2 | 60585806 | T | C | -0.0588 | 0.0064 | -9.1875 | 3.37E-20 | 933970 |
| T20D | rs2080385 | 2 | 65292762 | T | G | -0.0551 | 0.0074 | -7.445945946 | 1.24E-13 | 933970 |
| T21D | rs11688682 | 2 | 121347612 | C | G | -0.0581 | 0.0076 | -7.644736842 | 2.93E-14 | 933970 |
| T22D | rs7568172 | 2 | 158335340 | A | G | -0.0827 | 0.0135 | -6.125925926 | 9.19E-10 | 933970 |
| T23D | rs7572970 | 2 | 161136656 | A | G | -0.0447 | 0.0072 | -6.208333333 | 6.12E-10 | 933970 |
| T24D | rs1128249 | 2 | 165528624 | T | G | -0.0603 | 0.0065 | -9.276923077 | 1.56E-20 | 933970 |
| T25D | rs2972144 | 2 | 227101411 | A | G | -0.0911 | 0.0066 | -13.8030303 | 2.19E-43 | 933970 |
| T26D | rs11709077 | 3 | 12336507 | A | G | -0.104 | 0.0097 | -10.72164948 | 1.26E-26 | 933970 |
| T27D | rs1496653 | 3 | 23454790 | A | G | 0.0665 | 0.0079 | 8.417721519 | 2.49E-17 | 933970 |
| T28D | rs891368 | 3 | 53123273 | A | G | -0.0378 | 0.0064 | -5.90625 | 3.25E-09 | 933970 |
| T29D | rs2292662 | 3 | 63897215 | T | C | -0.0645 | 0.0088 | -7.329545455 | 2.24E-13 | 933970 |
| T30D | rs4132228 | 3 | 64708114 | T | C | -0.054 | 0.0071 | -7.605633803 | 3.34E-14 | 933970 |
| T31D | rs11708067 | 3 | 123065778 | A | G | 0.0882 | 0.0077 | 11.45454545 | 5.05E-30 | 933970 |
| T32D | rs9873519 | 3 | 124921457 | T | C | 0.0371 | 0.0064 | 5.796875 | 6.29E-09 | 933970 |
| T33D | rs62271373 | 3 | 150066540 | A | T | 0.0882 | 0.0144 | 6.125 | 1.03E-09 | 933970 |
| T34D | rs28663084 | 3 | 152382187 | A | G | -0.0376 | 0.0068 | -5.529411765 | 3.30E-08 | 933970 |
| T35D | rs6444809 | 3 | 168232423 | T | C | 0.0544 | 0.0096 | 5.666666667 | 1.63E-08 | 933970 |
| T36D | rs8192675 | 3 | 170724883 | T | C | 0.0659 | 0.007 | 9.414285714 | 5.77E-21 | 933970 |
| T37D | rs7633675 | 3 | 185510613 | T | G | -0.1085 | 0.0068 | -15.95588235 | 3.21E-57 | 933970 |
| T38D | rs3887925 | 3 | 186665645 | T | C | 0.0539 | 0.0065 | 8.292307692 | 1.01E-16 | 933970 |
| T39D | rs56187241 | 4 | 738275 | T | C | 0.1014 | 0.0157 | 6.458598726 | 1.07E-10 | 933970 |
| T40D | rs4865436 | 4 | 1788130 | C | G | -0.049 | 0.0075 | -6.533333333 | 8.04E-11 | 933970 |
| T41D | rs10937721 | 4 | 6306763 | C | G | 0.085 | 0.0066 | 12.87878788 | 5.39E-38 | 933970 |
| T42D | rs7667864 | 4 | 17811914 | A | C | -0.0402 | 0.0071 | -5.661971831 | 1.64E-08 | 933970 |
| T43D | rs13130484 | 4 | 45175691 | T | C | 0.0435 | 0.0067 | 6.492537313 | 8.49E-11 | 933970 |
| T44D | rs79920718 | 4 | 83578271 | A | G | 0.0389 | 0.0067 | 5.805970149 | 6.43E-09 | 933970 |
| T45D | rs6821438 | 4 | 95091911 | A | G | 0.0397 | 0.0064 | 6.203125 | 5.10E-10 | 933970 |
| T46D | rs10516495 | 4 | 103900090 | A | T | -0.0418 | 0.0069 | -6.057971014 | 1.46E-09 | 933970 |
| T47D | rs7669833 | 4 | 153513369 | A | T | -0.0572 | 0.007 | -8.171428571 | 3.52E-16 | 933970 |
| T48D | rs745805 | 4 | 185718132 | A | T | 0.0632 | 0.0084 | 7.523809524 | 4.52E-14 | 933970 |
| T49D | rs147581833 | 5 | 14755919 | T | C | -0.3713 | 0.0489 | -7.593047035 | 3.11E-14 | 933970 |
| T50D | rs6885132 | 5 | 14768092 | C | G | 0.077 | 0.011 | 7 | 2.49E-12 | 933970 |
| T51D | rs11958808 | 5 | 44945090 | C | G | 0.0403 | 0.0066 | 6.106060606 | 9.99E-10 | 933970 |
| T52D | rs702634 | 5 | 53271420 | A | G | 0.0503 | 0.0069 | 7.289855072 | 3.37E-13 | 933970 |
| T53D | rs459193 | 5 | 55806751 | A | G | -0.0722 | 0.0073 | -9.890410959 | 6.78E-23 | 933970 |
| T54D | rs34341 | 5 | 74934009 | A | T | -0.044 | 0.0065 | -6.769230769 | 1.22E-11 | 933970 |
| T55D | rs7732130 | 5 | 76435004 | A | G | -0.0606 | 0.007 | -8.657142857 | 5.69E-18 | 933970 |
| T56D | rs78408340 | 5 | 102338739 | C | G | -0.3788 | 0.0389 | -9.737789203 | 2.35E-22 | 933970 |
| T57D | rs115505614 | 5 | 102422968 | T | C | 0.1657 | 0.0149 | 11.12080537 | 7.58E-29 | 933970 |
| T58D | rs329122 | 5 | 133864599 | A | G | 0.0366 | 0.0065 | 5.630769231 | 1.72E-08 | 933970 |
| T59D | rs9379084 | 6 | 7231843 | A | G | -0.0994 | 0.0106 | -9.377358491 | 5.48E-21 | 933970 |
| T60D | rs9368222 | 6 | 20686996 | A | C | 0.1379 | 0.0071 | 19.42253521 | 1.42E-83 | 933970 |
| T61D | rs3094682 | 6 | 31264461 | A | C | -0.0619 | 0.0082 | -7.548780488 | 3.47E-14 | 933970 |
| T62D | rs601945 | 6 | 32573415 | A | G | -0.0849 | 0.0086 | -9.872093023 | 4.66E-23 | 933970 |
| T63D | rs6937438 | 6 | 43815364 | A | G | -0.0514 | 0.007 | -7.342857143 | 2.35E-13 | 933970 |
| T64D | rs3798519 | 6 | 50788778 | A | C | -0.0616 | 0.0082 | -7.512195122 | 4.60E-14 | 933970 |
| T65D | rs1665901 | 6 | 107433400 | A | T | 0.0398 | 0.0068 | 5.852941176 | 4.97E-09 | 933970 |
| T66D | rs11759026 | 6 | 126792095 | A | G | -0.066 | 0.0075 | -8.8 | 2.04E-18 | 933970 |
| T67D | rs1573090 | 6 | 137302159 | T | G | 0.0458 | 0.0065 | 7.046153846 | 1.72E-12 | 933970 |
| T68D | rs474513 | 6 | 160770312 | A | G | 0.0399 | 0.0064 | 6.234375 | 4.18E-10 | 933970 |
| T69D | rs4709746 | 6 | 164133001 | T | C | -0.0567 | 0.0096 | -5.90625 | 3.95E-09 | 933970 |
| T70D | rs17168486 | 7 | 14898282 | T | C | 0.0672 | 0.0083 | 8.096385542 | 4.50E-16 | 933970 |
| T71D | rs2215383 | 7 | 15062983 | T | C | -0.0641 | 0.0064 | -10.015625 | 1.06E-23 | 933970 |
| T72D | rs849134 | 7 | 28196222 | A | G | 0.0899 | 0.0064 | 14.046875 | 5.35E-45 | 933970 |
| T73D | rs917195 | 7 | 30728452 | T | C | -0.0473 | 0.0077 | -6.142857143 | 1.03E-09 | 933970 |
| T74D | rs878521 | 7 | 44255643 | A | G | 0.0618 | 0.0074 | 8.351351351 | 9.31E-17 | 933970 |
| T75D | rs11496066 | 7 | 102486254 | T | C | 0.0508 | 0.0083 | 6.120481928 | 8.17E-10 | 933970 |
| T76D | rs1562396 | 7 | 130457914 | A | G | -0.0555 | 0.0069 | -8.043478261 | 9.64E-16 | 933970 |
| T77D | rs1117610 | 7 | 150541189 | A | T | -0.0423 | 0.0076 | -5.565789474 | 3.13E-08 | 933970 |
| T78D | rs6459737 | 7 | 156975136 | A | G | -0.0583 | 0.0067 | -8.701492537 | 3.31E-18 | 933970 |
| T79D | rs11774915 | 8 | 9188762 | T | C | 0.0436 | 0.0068 | 6.411764706 | 1.49E-10 | 933970 |
| T80D | rs12680692 | 8 | 12618225 | A | T | 0.0413 | 0.0071 | 5.816901408 | 6.60E-09 | 933970 |
| T81D | rs7819706 | 8 | 19844415 | A | G | 0.0669 | 0.0101 | 6.623762376 | 2.82E-11 | 933970 |
| T82D | rs508419 | 8 | 41522991 | A | G | -0.0811 | 0.0075 | -10.81333333 | 5.43E-27 | 933970 |
| T83D | rs10097617 | 8 | 95961626 | T | C | 0.0487 | 0.0064 | 7.609375 | 2.44E-14 | 933970 |
| T84D | rs3802177 | 8 | 118185025 | A | G | -0.1077 | 0.0069 | -15.60869565 | 9.16E-55 | 933970 |
| T85D | rs17772814 | 8 | 128711742 | A | G | -0.0746 | 0.0125 | -5.968 | 2.13E-09 | 933970 |
| T86D | rs1561927 | 8 | 129568078 | T | C | -0.042 | 0.0072 | -5.833333333 | 6.12E-09 | 933970 |
| T87D | rs4977213 | 8 | 145507304 | T | C | -0.0507 | 0.0069 | -7.347826087 | 2.19E-13 | 933970 |
| T88D | rs12719778 | 8 | 145879883 | T | C | 0.0381 | 0.0065 | 5.861538462 | 4.38E-09 | 933970 |
| T89D | rs672271 | 9 | 3273781 | T | C | -0.06 | 0.011 | -5.454545455 | 4.83E-08 | 933970 |
| T90D | rs10974438 | 9 | 4291928 | A | C | -0.0514 | 0.0067 | -7.671641791 | 1.71E-14 | 933970 |
| T91D | rs62563593 | 9 | 19065825 | A | G | -0.0394 | 0.0065 | -6.061538462 | 1.28E-09 | 933970 |
| T92D | rs2383205 | 9 | 22060935 | A | G | -0.0533 | 0.0065 | -8.2 | 2.20E-16 | 933970 |
| T93D | rs10811660 | 9 | 22134068 | A | G | -0.1598 | 0.0086 | -18.58139535 | 2.54E-77 | 933970 |
| T94D | rs1412234 | 9 | 28410683 | T | C | -0.0393 | 0.0068 | -5.779411765 | 7.71E-09 | 933970 |
| T95D | rs12001437 | 9 | 34074476 | T | C | -0.0402 | 0.0066 | -6.090909091 | 1.10E-09 | 933970 |
| T96D | rs17791513 | 9 | 81905590 | A | G | 0.1016 | 0.0132 | 7.696969697 | 1.35E-14 | 933970 |
| T97D | rs2796441 | 9 | 84308948 | A | G | -0.0674 | 0.0065 | -10.36923077 | 2.97E-25 | 933970 |
| T98D | rs55653563 | 9 | 97001682 | A | C | 0.0435 | 0.0072 | 6.041666667 | 1.73E-09 | 933970 |
| T99D | rs505922 | 9 | 136149229 | T | C | -0.0473 | 0.0068 | -6.955882353 | 3.65E-12 | 933970 |
| T100D | rs28533815 | 9 | 139241828 | T | C | -0.0748 | 0.008 | -9.35 | 5.45E-21 | 933970 |
| T101D | rs7077792 | 10 | 12316768 | T | C | 0.0823 | 0.0081 | 10.16049383 | 1.82E-24 | 933970 |
| T102D | rs2812545 | 10 | 71339737 | A | G | 0.0413 | 0.0065 | 6.353846154 | 1.99E-10 | 933970 |
| T103D | rs1381937 | 10 | 71464834 | A | C | 0.0442 | 0.0064 | 6.90625 | 4.50E-12 | 933970 |
| T104D | rs703972 | 10 | 80952826 | C | G | -0.0698 | 0.0065 | -10.73846154 | 5.77E-27 | 933970 |
| T105D | rs10882099 | 10 | 94460650 | T | C | 0.1095 | 0.0065 | 16.84615385 | 7.73E-64 | 933970 |
| T106D | rs116425039 | 10 | 114681965 | A | G | -0.272 | 0.0348 | -7.816091954 | 5.08E-15 | 933970 |
| T107D | rs146262265 | 10 | 114738287 | T | C | 0.2255 | 0.0399 | 5.651629073 | 1.58E-08 | 933970 |
| T108D | rs4267006 | 10 | 114758779 | T | G | 0.2922 | 0.0076 | 38.44736842 | 9.05e-320 | 933970 |
| T109D | rs35198330 | 10 | 114844784 | T | G | -0.0938 | 0.0149 | -6.295302013 | 2.81E-10 | 933970 |
| T110D | rs189966089 | 10 | 114886341 | T | C | -0.1284 | 0.0221 | -5.809954751 | 6.17E-09 | 933970 |
| T111D | rs1045216 | 10 | 124189197 | A | G | 0.0369 | 0.0065 | 5.676923077 | 1.31E-08 | 933970 |
| T112D | rs4929965 | 11 | 2197286 | A | G | 0.0668 | 0.0068 | 9.823529412 | 9.67E-23 | 933970 |
| T113D | rs11023936 | 11 | 2755552 | T | C | 0.039 | 0.0071 | 5.492957746 | 4.31E-08 | 933970 |
| T114D | rs2237895 | 11 | 2857194 | A | C | -0.0892 | 0.0066 | -13.51515152 | 1.15E-41 | 933970 |
| T115D | rs141521721 | 11 | 14763828 | A | C | 0.1212 | 0.0214 | 5.663551402 | 1.39E-08 | 933970 |
| T116D | rs5215 | 11 | 17408630 | T | C | -0.0706 | 0.0066 | -10.6969697 | 9.85E-27 | 933970 |
| T117D | rs145678014 | 11 | 32927778 | T | G | -0.1048 | 0.0163 | -6.429447853 | 1.40E-10 | 933970 |
| T118D | rs2767036 | 11 | 34982148 | A | C | -0.0389 | 0.007 | -5.557142857 | 2.94E-08 | 933970 |
| T119D | rs1061810 | 11 | 43877934 | A | C | 0.0502 | 0.007 | 7.171428571 | 8.31E-13 | 933970 |
| T120D | rs1783541 | 11 | 65294799 | T | C | 0.0608 | 0.0081 | 7.50617284 | 4.65E-14 | 933970 |
| T121D | rs77464186 | 11 | 72460398 | A | C | 0.1002 | 0.0087 | 11.51724138 | 9.30E-31 | 933970 |
| T122D | rs10830963 | 11 | 92708710 | C | G | -0.101 | 0.0071 | -14.22535211 | 1.12E-45 | 933970 |
| T123D | rs3019208 | 11 | 93061493 | A | G | 0.0397 | 0.0068 | 5.838235294 | 5.43E-09 | 933970 |
| T124D | rs9665898 | 11 | 128043666 | T | C | -0.0525 | 0.0094 | -5.585106383 | 2.53E-08 | 933970 |
| T125D | rs10750397 | 11 | 128234144 | A | G | 0.0394 | 0.0071 | 5.549295775 | 3.13E-08 | 933970 |
| T126D | rs67232546 | 11 | 128398938 | T | C | 0.0531 | 0.008 | 6.6375 | 2.51E-11 | 933970 |
| T127D | rs11063029 | 12 | 4301301 | T | C | 0.0858 | 0.0138 | 6.217391304 | 5.36E-10 | 933970 |
| T128D | rs11063069 | 12 | 4374373 | A | G | -0.056 | 0.0079 | -7.088607595 | 9.94E-13 | 933970 |
| T129D | rs76895963 | 12 | 4384844 | T | G | 0.4826 | 0.0275 | 17.54909091 | 9.12E-69 | 933970 |
| T130D | rs10842675 | 12 | 26259919 | A | G | 0.0381 | 0.0067 | 5.686567164 | 1.30E-08 | 933970 |
| T131D | rs7966976 | 12 | 27962934 | A | G | -0.0751 | 0.0081 | -9.271604938 | 1.22E-20 | 933970 |
| T132D | rs2258238 | 12 | 66221060 | A | T | -0.102 | 0.0106 | -9.622641509 | 5.14E-22 | 933970 |
| T133D | rs1705263 | 12 | 71523043 | A | C | -0.0484 | 0.0064 | -7.5625 | 3.51E-14 | 933970 |
| T134D | rs77864822 | 12 | 97848775 | A | G | 0.0753 | 0.0129 | 5.837209302 | 5.01E-09 | 933970 |
| T135D | rs1426371 | 12 | 108629780 | A | G | -0.0517 | 0.0073 | -7.082191781 | 1.74E-12 | 933970 |
| T136D | rs7313918 | 12 | 118394008 | T | C | -0.0559 | 0.0094 | -5.946808511 | 2.99E-09 | 933970 |
| T137D | rs56348580 | 12 | 121432117 | C | G | -0.0617 | 0.0069 | -8.942028986 | 4.31E-19 | 933970 |
| T138D | rs35318451 | 12 | 133068484 | A | G | 0.0478 | 0.007 | 6.828571429 | 9.50E-12 | 933970 |
| T139D | rs11842871 | 13 | 31042452 | T | G | -0.04 | 0.0073 | -5.479452055 | 4.83E-08 | 933970 |
| T140D | rs576674 | 13 | 33554302 | A | G | -0.0538 | 0.0086 | -6.255813953 | 3.70E-10 | 933970 |
| T141D | rs9563615 | 13 | 59077406 | A | T | 0.0408 | 0.0072 | 5.666666667 | 1.63E-08 | 933970 |
| T142D | rs1359790 | 13 | 80717156 | A | G | -0.0817 | 0.0071 | -11.50704225 | 1.76E-30 | 933970 |
| T143D | rs7325671 | 13 | 108797836 | T | C | 0.0545 | 0.0096 | 5.677083333 | 1.53E-08 | 933970 |
| T144D | rs17122772 | 14 | 23288935 | C | G | -0.0428 | 0.0077 | -5.558441558 | 3.31E-08 | 933970 |
| T145D | rs17522122 | 14 | 33302882 | T | G | 0.0356 | 0.0064 | 5.5625 | 2.49E-08 | 933970 |
| T146D | rs8008910 | 14 | 79944099 | A | G | 0.0554 | 0.0076 | 7.289473684 | 4.22E-13 | 933970 |
| T147D | rs2896177 | 14 | 91968313 | A | G | -0.0367 | 0.0064 | -5.734375 | 9.13E-09 | 933970 |
| T148D | rs3783394 | 14 | 103858673 | A | G | -0.038 | 0.0067 | -5.671641791 | 1.42E-08 | 933970 |
| T149D | rs34715063 | 15 | 38873115 | T | C | -0.0772 | 0.0099 | -7.797979798 | 8.40E-15 | 933970 |
| T150D | rs2277536 | 15 | 41857303 | T | C | -0.0429 | 0.007 | -6.128571429 | 9.63E-10 | 933970 |
| T151D | rs11856307 | 15 | 62399093 | A | C | 0.047 | 0.0065 | 7.230769231 | 4.48E-13 | 933970 |
| T152D | rs4984251 | 15 | 63889022 | A | G | -0.0375 | 0.0064 | -5.859375 | 4.32E-09 | 933970 |
| T153D | rs12910361 | 15 | 77782335 | A | G | -0.0814 | 0.007 | -11.62857143 | 3.95E-31 | 933970 |
| T154D | rs2351707 | 15 | 90384045 | T | C | -0.0644 | 0.007 | -9.2 | 4.30E-20 | 933970 |
| T155D | rs113343095 | 15 | 91522092 | A | G | 0.066 | 0.0093 | 7.096774194 | 1.41E-12 | 933970 |
| T156D | rs6600191 | 16 | 295795 | T | C | 0.0587 | 0.0085 | 6.905882353 | 4.47E-12 | 933970 |
| T157D | rs12325539 | 16 | 30033633 | T | C | -0.041 | 0.0065 | -6.307692308 | 2.69E-10 | 933970 |
| T158D | rs17817964 | 16 | 53828066 | T | C | 0.1196 | 0.0065 | 18.4 | 8.44E-76 | 933970 |
| T159D | rs13330951 | 16 | 69563842 | A | G | 0.0361 | 0.0064 | 5.640625 | 1.58E-08 | 933970 |
| T160D | rs72802358 | 16 | 75243657 | C | G | -0.1135 | 0.0107 | -10.60747664 | 2.22E-26 | 933970 |
| T161D | rs2925979 | 16 | 81534790 | T | C | 0.0546 | 0.007 | 7.8 | 7.07E-15 | 933970 |
| T162D | rs12920022 | 16 | 89564055 | A | T | 0.0528 | 0.0092 | 5.739130435 | 1.00E-08 | 933970 |
| T163D | rs8071043 | 17 | 3988451 | T | C | -0.0523 | 0.0068 | -7.691176471 | 1.53E-14 | 933970 |
| T164D | rs4925115 | 17 | 17721457 | A | G | 0.0399 | 0.0067 | 5.955223881 | 2.61E-09 | 933970 |
| T165D | rs2107133 | 17 | 36064897 | A | G | 0.0643 | 0.0097 | 6.628865979 | 4.02E-11 | 933970 |
| T166D | rs10908278 | 17 | 36099952 | A | T | -0.0749 | 0.0067 | -11.17910448 | 5.25E-29 | 933970 |
| T167D | rs2062213 | 17 | 40566750 | C | G | -0.0743 | 0.012 | -6.191666667 | 6.80E-10 | 933970 |
| T168D | rs35895680 | 17 | 47060322 | A | C | -0.0554 | 0.0069 | -8.028985507 | 1.08E-15 | 933970 |
| T169D | rs58642235 | 17 | 62202689 | T | C | -0.0566 | 0.0094 | -6.021276596 | 1.90E-09 | 933970 |
| T170D | rs2080090 | 17 | 65828371 | A | T | 0.0526 | 0.0084 | 6.261904762 | 3.39E-10 | 933970 |
| T171D | rs7240767 | 18 | 7070642 | T | C | -0.0372 | 0.0066 | -5.636363636 | 1.71E-08 | 933970 |
| T172D | rs1431841 | 18 | 40087098 | T | G | 0.0429 | 0.0077 | 5.571428571 | 3.08E-08 | 933970 |
| T173D | rs8097210 | 18 | 57857135 | T | G | -0.0537 | 0.0072 | -7.458333333 | 1.06E-13 | 933970 |
| T174D | rs79688165 | 18 | 58050968 | T | C | -0.1383 | 0.0226 | -6.119469027 | 9.64E-10 | 933970 |
| T175D | rs4804833 | 19 | 7970635 | A | G | 0.048 | 0.0066 | 7.272727273 | 3.42E-13 | 933970 |
| T176D | rs10419627 | 19 | 13036677 | A | G | 0.0431 | 0.0065 | 6.630769231 | 3.15E-11 | 933970 |
| T177D | rs739846 | 19 | 19419071 | A | G | 0.0877 | 0.0119 | 7.369747899 | 2.02E-13 | 933970 |
| T178D | rs3786900 | 19 | 33897149 | A | G | 0.0434 | 0.0072 | 6.027777778 | 1.89E-09 | 933970 |
| T179D | rs429358 | 19 | 45411941 | T | C | 0.0746 | 0.0091 | 8.197802198 | 2.62E-16 | 933970 |
| T180D | rs10406431 | 19 | 46157019 | A | G | 0.0603 | 0.0065 | 9.276923077 | 1.56E-20 | 933970 |
| T181D | rs17744783 | 20 | 21460433 | A | T | 0.0577 | 0.0103 | 5.601941748 | 1.87E-08 | 933970 |
| T182D | rs1007090 | 20 | 32582871 | T | C | -0.044 | 0.0068 | -6.470588235 | 1.01E-10 | 933970 |
| T183D | rs1800961 | 20 | 43042364 | T | C | 0.1602 | 0.0175 | 9.154285714 | 5.10E-20 | 933970 |
| T184D | rs1999536 | 20 | 45581777 | C | G | -0.0404 | 0.0065 | -6.215384615 | 4.86E-10 | 933970 |
| T185D | rs11699802 | 20 | 48832135 | T | C | -0.0443 | 0.0065 | -6.815384615 | 8.84E-12 | 933970 |
| T186D | rs4812034 | 20 | 57397566 | T | G | 0.0416 | 0.0064 | 6.5 | 7.35E-11 | 933970 |
| T187D | rs2023681 | 22 | 30599562 | A | G | -0.0826 | 0.0115 | -7.182608696 | 7.40E-13 | 933970 |
| T188D | rs36138276 | 22 | 50422348 | A | G | -0.0431 | 0.0066 | -6.53030303 | 6.40E-11 | 933970 |
